# From Product Disclosure to Brand Trust: How Voluntary Negative-Attribute Disclosure Shapes Cross-Product Responses in Digital Food Interfaces

**DOI:** 10.3390/bs16081410

**Published:** 2026-08-17

**Authors:** Xing Meng, Jinxi Ji, Cong Sun

**Affiliations:** 1 School of Business, Ningbo University, Ningbo 315211, China; mengxing@nbu.edu.cn; 2 Pan Tianshou College of Architecture, Art and Design, Ningbo University, Ningbo 315211, China; 2311370004@nbu.edu.cn

**Keywords:** digital food interfaces, voluntary negative-attribute disclosure, brand trust, cross-product trust transfer, cognitive trust, affective trust, disclosure unexpectedness, product fit

## Abstract

Digital food interfaces increasingly enable brands to foreground decision-relevant information at the point of choice, including attributes that may disadvantage a focal product. This emerging practice raises a counterintuitive question: can voluntarily revealing a negative attribute of Product A build brand trust that shapes responses to Product B? Across three online experiments, we examine the cross-product effects of voluntary negative-attribute disclosure, the parallel roles of cognitive and affective trust, and the boundary conditions of disclosure unexpectedness and product fit. Studies 1 and 2 showed that voluntary negative-attribute disclosure increased target-product trust relative to self-promotion. Study 1 further identified cognitive and affective trust as parallel brand-level pathways to target-product trust and purchase intention. Objective description produced comparable downstream responses, while voluntary negative-attribute disclosure generated stronger cognitive trust, revealing a distinctive pattern of trust formation. Study 2 showed that high unexpectedness weakened both pathways, yet affective trust retained downstream influence as the cognitive pathway attenuated. Study 3 showed that under low product fit, both pathways continued to support purchase intention, while their translation into target-product trust was constrained. Together, the findings extend transparency research from focal-product effects to cross-product consequences and show how voluntarily foregrounding an unfavorable product attribute can transform a product-level disclosure into brand-level trust with implications across a product portfolio.

## 1. Introduction

In contemporary Chinese digital food markets, brands are increasingly integrating health-related product information into the interfaces through which consumers evaluate and order products. McDonald’s China illustrates this development: since 2019, it has promoted 500 kcal meal combinations through online ordering platforms, where calorie counts are prominently displayed alongside individual meal offerings at the point of choice; its website also provides nutrition information for regular menu items through an online nutrition calculator (McDonald’s China, 2019, 2025). Similar practices are increasingly visible in beverage categories. HEYTEA uses a caffeine traffic-light system directly in its mini-program ordering interface, visually classifying products by caffeine level and pairing red-light items with consumption guidance such as a recommendation to drink them before midday (Meng, 2024). CHAGEE similarly foregrounds calorie and caffeine information on its digital product pages, displaying numerical values alongside a color-coded caffeine gauge that situates each drink within a graduated range before purchase (Xu, 2026). Together, these initiatives illustrate an emerging commercial development in which decision-relevant product information is not merely available but increasingly made visually salient at the point of choice. Digital food interfaces provide the infrastructure that makes this development easier to implement and extend across a brand portfolio: their expandable information architecture, relatively low updating and replication costs, integration with browsing and ordering, and portfolio-linked navigation allow information to be displayed, revised, and repeated across multiple products within the same branded environment (Olzenak et al., 2020; Pomeranz et al., 2022; Sharib et al., 2024; Volpe et al., 2026; Yang et al., 2025).

Within this emerging practice, voluntary negative-attribute disclosure represents a particularly consequential communication choice. It occurs when a brand foregrounds an unfavorable but decision-relevant attribute—such as high sugar, sodium, caffeine, or calorie content—rather than limiting the interface to favorable claims. The visibility of such information directs attention to a credibility question: what does the brand’s decision to foreground an unfavorable attribute reveal about the brand as an information provider? Because such disclosure may conflict with the brand’s immediate persuasive interests, consumers may use it to infer the brand’s reliability, honesty, and concern for consumers (Eisend, 2006; Fennis & Stroebe, 2014; Park & Yi, 2023). This leads to the central theoretical question of the present research: whether consumers treat voluntary negative-attribute disclosure only as local information about Product A or elevate it into brand-level trust that shapes their responses to Product B.

Existing research provides important foundations for this question. Digital consumer behavior research highlights the role of information quality, perceived risk, and trust in online decision making (Kim et al., 2008; Lamberton & Stephen, 2016; Han et al., 2023), while brand transparency research shows that consumers use disclosure practices to infer credibility, integrity, and trustworthiness (Cambier & Poncin, 2020; Montecchi et al., 2024; Sansome et al., 2024, 2025). Much of this work examines either system-level transparency or responses to the disclosed object itself. Sun et al. (2025) provide the direct starting point for the present research by showing that voluntary disclosure of an unhealthy product attribute can increase perceived transparency and trust toward the disclosed product. The present research moves from focal-product effect to cross-product transfer: it examines whether disclosure about Product A shapes cognitive and affective trust in the brand and consequently influences trust in and purchase intention toward Product B. This shift connects product-level transparency with brand extension and spillover research and asks how brand-level trust is formed, under what conditions it remains operative, and how it translates across products within the same brand portfolio (Aaker & Keller, 1990; C. Peng et al., 2023; Roehm & Tybout, 2006).

Building on signaling theory, trust theory, and research on brand-related transfer, we propose that such product-level disclosure may generate two related but distinguishable forms of brand trust. Cognitive trust reflects judgments that the brand is reliable, honest, and dependable, whereas affective trust reflects judgments that the brand is sincere, benevolent, and concerned with consumers’ interests (Johnson & Grayson, 2005; Mayer et al., 1995). Because these judgments concern the brand rather than only the disclosed product, they may influence responses to another product offered under the same brand. We examine two downstream responses separately: trust in the target product, a relatively proximal evaluative response to the transferred brand inference, and purchase intention toward the target product, a more distal and integrative decision intention that may also depend on product preferences, situational needs, and other choice considerations.

Against this background, the research addresses four questions:

- RQ1. Does a brand’s voluntary disclosure of an unfavorable product attribute in a digital food interface influence consumers’ trust in and purchase intention toward another product under the same brand, relative to self-promotional disclosure, objective description, and no prior Product A exposure?
- RQ2. Are the cross-product effects of voluntary negative-attribute disclosure transmitted through cognitive trust and affective trust in the brand?
- RQ3. How does the unexpectedness of the disclosed negative attribute alter the operation of the cognitive and affective trust pathways?
- RQ4. Under low product fit, do disclosure-generated cognitive and affective trust continue to support purchase intention toward the target product while their transmission to target-product trust becomes constrained?

We address these questions through a three-study sequence that progressively examines how disclosure-based brand trust emerges, persists, and translates across products. Study 1 tests whether voluntary negative-attribute disclosure about Product A shapes target-product trust and purchase intention toward Product B and examines cognitive and affective trust as parallel brand-level pathways. Study 2 then investigates how the unexpectedness of the disclosed negative attribute changes the formation and downstream transmission of those pathways. Study 3 turns to the receiving side of the transfer process by examining how disclosure-generated brand trust translates into target-product trust and purchase intention when product fit is low versus high. Together, the studies explain how voluntary negative-attribute disclosure about one product can acquire brand-level meaning and shape consumers’ responses to another product under the same brand.

## 2. Theoretical Background and Hypotheses

### 2.1. Product-Level Transparency in Digital Food Interfaces

Digital food interfaces—including online menus, food-ordering applications, brand-operated mini-programs, and digital grocery platforms—provide an expandable and updatable infrastructure for product-level disclosure at the point of choice. Their layered information architecture and portfolio-wide updating capacity relax fixed physical space and revision constraints, while their integration with browsing, comparison, customization, and ordering places decision-relevant information directly within the consumer choice process. Low-friction movement between product pages also gives information encountered for Product A a practical opportunity to remain accessible when consumers subsequently evaluate Product B. These features make salient and repeatable disclosure across a brand portfolio more feasible than under many fixed physical formats (Olzenak et al., 2020; Pomeranz et al., 2022; Sharib et al., 2024; Volpe et al., 2026).

Within this environment, product-level transparency should be distinguished from mere information availability. Product information may be technically present but difficult to locate, interpret, compare, or attribute to an intentional communication choice by the brand. Product-level transparency instead concerns whether decision-relevant attributes are made sufficiently visible and interpretable for consumers to recognize that the brand has chosen to communicate them. Brand transparency research similarly distinguishes the availability of information from consumers’ perceptions that a brand communicates openly, clearly, and accountably (Cambier & Poncin, 2020; Montecchi et al., 2024; Sansome et al., 2024). Recent evidence further shows that consumers vary substantially in whether and why they engage with transparency information, and that such information becomes particularly salient when it is personally relevant or useful for managing decision risk (Sansome et al., 2025).

This distinction is important because consumers may infer more than the literal meaning of a disclosed product attribute. In a branded digital interface, descriptions, nutrition labels, warnings, claims, and attribute disclosures are also observable communication choices. Consumers may therefore judge not only what a product is like but also what kind of information provider the brand appears to be. When product information appears credible and attributable to the brand’s own disclosure decision, it can provide a basis for broader judgments of brand reliability and integrity (Cambier & Poncin, 2020; Kim et al., 2008; Montecchi et al., 2024). The central issue addressed in this research is whether such a product-level disclosure cue remains attached to the disclosed product or acquires brand-level meaning that influences consumers’ responses to another product under the same brand.

Voluntary negative-attribute disclosure provides the focal empirical setting through which we examine this possibility. It represents a consequential disclosure choice because the brand actively draws attention to information that is unfavorable but relevant to consumer decision making. The following section develops why such a disclosure can invite a broader inference about the brand and influence responses beyond the focal product.

### 2.2. Voluntary Negative-Attribute Disclosure as a Potentially Diagnostic Product-Level Signal

Voluntary negative-attribute disclosure refers to a brand’s intentional communication of an unfavorable but decision-relevant product attribute, such as high sugar, sodium, caffeine, or calorie content. Its theoretical relevance lies in the brand’s decision to foreground information that may conflict with its immediate persuasive interest. Unfavorable product information can reduce evaluations of the disclosed product when it signals risk, inferior performance, or a mismatch with consumer goals; at the same time, the decision to communicate that information may reveal how the brand approaches the provision of decision-relevant information.

Signaling theory proposes that, under information asymmetry, communication becomes more credible when it is difficult to reconcile with purely self-serving persuasion or when sending it may involve some disadvantage to the sender (Connelly et al., 2011; Erdem & Swait, 2004; Kirmani & Rao, 2000). Research on two-sided communication likewise shows that acknowledging unfavorable information can increase source credibility relative to uniformly positive communication because the message appears more balanced and less one-sided (Eisend, 2006). Related research finds that voluntary company or product self-disclosure can soften negative reactions or strengthen trust when consumers interpret the disclosure as open rather than evasive or manipulative (Fennis & Stroebe, 2014; Jahn & Brühl, 2019; Park & Yi, 2023). More recently, three experimental studies by Sansome et al. (2026) showed that disclosures acknowledging negative brand activities were perceived as more transparent than positive disclosures, although their downstream consequences depended on contextual factors such as prior brand credibility. Taken together, this literature suggests that voluntary negative-attribute disclosure can function as a potentially diagnostic product-level signal: the act of disclosing may invite an inference that the brand is willing to communicate information that consumers need, even when the information is not uniformly favorable.

This inference should be especially relevant when voluntary negative-attribute disclosure is compared with self-promotional disclosure. Self-promotional disclosure highlights favorable product attributes and is consistent with ordinary expectations of marketplace persuasion. It may communicate useful product information, but it provides limited evidence that the brand is willing to depart from selective positive presentation. Voluntary negative-attribute disclosure, by contrast, makes a less favorable attribute salient and may therefore provide a stronger basis for judging the brand as a reliable and consumer-oriented information provider. A similar distinction applies to the no-prior-Product-A-exposure baseline. Participants who have not encountered Product A receive no prior disclosure choice from which to infer how the brand communicates decision-relevant product information. Voluntary negative-attribute disclosure supplies such a cue and may consequently affect how consumers respond to Product B.

Prior research provides an important focal-product point of departure. Sun et al. (2025) found that voluntarily disclosing unhealthy attributes in a digital food-ordering context could enhance perceived transparency and trust toward the disclosed product. The present research extends that framework by examining whether the resulting inference travels beyond the focal product. Brand extension and spillover research shows that associations formed around one branded offering can affect evaluations of another offering when consumers recognize a meaningful connection through the shared brand (Aaker & Keller, 1990; C. Peng et al., 2023; Roehm & Tybout, 2006). If voluntary negative-attribute disclosure changes consumers’ judgments of the brand as an information provider rather than merely informing them about Product A, the resulting inference should remain accessible when they evaluate Product B.

We therefore expect voluntary negative-attribute disclosure to produce more favorable cross-product responses than self-promotional disclosure and the absence of prior Product A exposure.

**H1.**  *Compared with self-promotional disclosure and no prior Product A exposure, voluntary negative-attribute disclosure in a digital food interface will increase consumers’ trust in and purchase intention toward another product within the same brand portfolio*.

### 2.3. Cognitive and Affective Trust as Parallel Brand-Level Pathways

For a product-level disclosure to influence another product, the inference generated by the disclosure must become associated with the brand rather than remain confined to Product A. Brand trust provides this cross-product bridge. Trust is not a unitary construct. Prior research distinguishes cognitive trust, which is grounded in judgments of competence, reliability, integrity, and dependability, from affective trust, which is grounded in perceived sincerity, benevolence, care, and relational goodwill (Chaudhuri & Holbrook, 2001; Johnson & Grayson, 2005; Mayer et al., 1995). This distinction is important for the present research because consumers may interpret the same disclosure decision in two psychologically different ways. The disclosure can lead consumers to believe that the brand is a reliable information provider, and it can also lead them to feel that the brand is sincere and consumer-oriented.

The cognitive trust pathway reflects consumers’ reasoned evaluation of the brand’s reliability and integrity. When consumers interpret the disclosure as evidence of reliable information provision, they may conclude that the brand is less likely to conceal decision-relevant information and more likely to provide balanced product communication. This inference can reduce uncertainty about the brand as an information source and increase confidence that other products under the same brand are also presented in a reliable manner. Cognitive trust is therefore expected to mediate trust transfer because it converts the informational meaning of the disclosure into a belief that the brand is dependable across products. This reasoning is consistent with trust theory and signaling research, which suggest that credible signals can reduce uncertainty and strengthen perceived reliability when consumers lack complete information (Akerlof, 1970; Connelly et al., 2011; Kim et al., 2008; Kirmani & Rao, 2000; Wu et al., 2023).

The affective trust pathway reflects consumers’ relational interpretation of the same disclosure decision. A brand that appears willing to communicate candidly may be perceived not only as reliable but also as sincere, benevolent, and respectful of consumers. This perception can generate a warmer relational response because consumers may interpret the brand’s communication style as evidence of goodwill rather than mere persuasion. Prior research shows that affective trust develops when consumers perceive sincerity, care, and benevolence in a relationship partner and that brand self-disclosure can foster intimacy and trust when it appears sincere rather than manipulative (Delgado-Ballester & Munuera-Alemán, 2005; Huaman-Ramirez et al., 2022; Johnson & Grayson, 2005; Tian et al., 2023). Affective trust is therefore expected to mediate trust transfer because it converts the relational meaning of the disclosure into a feeling that the brand has consumers’ interests in mind.

Both routes should support cross-product effects because they attach the informational and relational meaning of the disclosure to the brand rather than confining it to the disclosed product. Related digital commerce research has likewise emphasized that trust can translate into purchase-related responses in platform-mediated settings (Chen et al., 2022; Choi et al., 2024; Li & Huang, 2024). Trust transfer research suggests that trust formed in one object can influence evaluations of another when consumers perceive a meaningful connection between them (Konuk, 2020; Lim et al., 2006; Raza et al., 2023; Zhao et al., 2019). In the present context, the shared brand provides this connection. Accordingly, voluntary negative-attribute disclosure should influence trust in and purchase intention toward another product through parallel cognitive and affective trust pathways.

**H2.**  *Compared with self-promotional disclosure, the positive effect of voluntary negative-attribute disclosure on consumers’ trust in and purchase intention toward another product under the same brand will be mediated through two parallel brand-level pathways: cognitive trust in the brand and affective trust in the brand*.

### 2.4. Disclosure Unexpectedness and Trust Pathway Persistence

The meaning consumers assign to voluntary negative-attribute disclosure may depend on whether the disclosed drawback is expected for the product category. When an unfavorable attribute is relatively expected, it can be integrated into existing category knowledge with limited surprise. When the same type of drawback is unexpected, it creates a larger discrepancy between prior beliefs and the newly disclosed information. Unexpectedness is therefore not inherently favorable or unfavorable. Rather, it can simultaneously increase attention to the undesirable attribute and prompt consumers to consider why the brand chose to disclose it.

Expectancy-inconsistent information generally receives greater attention and requires more interpretive processing because it does not fit readily within an existing schema (Meyers-Levy & Tybout, 1989). In a product context, an unexpected negative attribute may heighten perceived risk and make consumers less willing to treat the information as routine (Dawar & Pillutla, 2000). Attribution theory further suggests that unexpected actions encourage observers to search for causal explanations and infer the actor’s underlying motives (Folkes, 1984). Persuasion knowledge research similarly indicates that consumers evaluate why marketers present information and whether a communication choice reflects genuine openness or strategic impression management (Darke & Ritchie, 2007; Friestad & Wright, 1994).

These two consequences create different implications for the cognitive and affective trust pathways. Cognitive trust depends on consumers’ ability to form a sufficiently stable judgment that the brand is reliable, dependable, and consistent as an information provider. A highly unexpected drawback can make the negative product implication more salient and difficult to reconcile with prior category expectations. The disclosure may consequently raise uncertainty about the product and weaken the extent to which the act of disclosure is translated into a broad reliability judgment. Thus, high unexpectedness should attenuate the cognitive trust pathway.

Affective trust may also be reduced when the disclosed drawback becomes more concerning; however, it can remain operative for a different reason. Even when consumers react negatively to the surprising attribute itself, the brand’s willingness to disclose that attribute may continue to convey relational information about sincerity and concern for informed consumer choice.

Therefore, high unexpectedness is expected to weaken the formation of disclosure-based trust generally. The cognitive pathway should be more vulnerable to losing its operative role, whereas the affective pathway may continue to function because it rests partly on the perceived sincerity conveyed by the act of disclosure.

**H3.**  *When the disclosed negative attribute is highly unexpected, the cognitive trust pathway linking voluntary negative-attribute disclosure to cross-product consumer responses will be attenuated, whereas the affective trust pathway will remain operative*.

### 2.5. Product Fit and the Translation of Brand Trust into Target-Product Responses

Product fit refers to the perceived relatedness, similarity, or coherence between the disclosed product and the target product. In brand extension research, fit is central because it determines whether consumers regard associations from one product as relevant to another product under the same brand (Aaker & Keller, 1990; Bridges et al., 2000; C. Peng et al., 2023; Völckner & Sattler, 2006). A recent systematic review of 102 brand extension studies shows that perceived fit is multidimensional and may involve shared physical characteristics, manufacturing capabilities, consumption situations, target users, complementary functions, or broader conceptual associations (Biallowons et al., 2026). High fit generally makes it easier to apply existing brand and product associations to the target offering, whereas low fit weakens the perceived relevance of those associations.

In the present framework, product fit conditions how brand-level trust is translated into two distinct target-product responses. Trust in the target product is a relatively proximal evaluative judgment: it concerns whether Product B itself appears reliable, credible, and worthy of confidence. Forming that judgment requires a basis for applying the disclosure-related brand inference to the specific product being evaluated. Under high fit, shared product characteristics, usage goals, or capabilities can supply such a basis. Under low fit, however, consumers have less category-relevant evidence to conclude that trust elicited by Product A disclosure establishes the reliability of Product B. Both cognitive and affective brand trust may therefore fail to translate reliably into target-product trust when the two products are weakly connected.

Purchase intention is conceptually different. It is a more distal and integrative decision intention rather than a direct measure of confidence in Product B. Purchase intention can be informed by trust but also by perceived value, need, preferences, situational goals, price, available alternatives, and perceived behavioral feasibility (Ajzen, 1991; Kim et al., 2008; Lim et al., 2006). The two outcomes therefore impose different inferential requirements and may exhibit different patterns. A consumer may hold a favorable brand-level orientation without possessing strong category-specific evidence about Product B; conversely, the consumer may trust Product B but remain unwilling to purchase it because the product does not match personal preferences or immediate needs.

Under low fit, disclosure-generated brand trust may therefore retain relevance for purchase intention even when it is insufficient to establish target-product trust. Cognitive trust in the brand can support a general willingness to transact with a brand perceived as reliable, while affective trust can support willingness to choose a brand perceived as sincere and consumer-oriented. These brand-level dispositions may contribute to purchase intention without requiring consumers to infer that Product A and Product B possess the same attributes, production capabilities, or product-specific reliability. The proposed divergence is specific to low-fit conditions and follows from the different inferential requirements of the two outcomes.

Accordingly, H4 focuses on the expected configuration of the two trust pathways under low product fit. When the disclosed and target products are weakly related, both forms of brand trust may continue to support purchase intention, but neither should provide a consistently sufficient basis for confidence in the target product itself.

**H4.**  *When product fit is low, the cognitive and affective trust pathways generated by voluntary negative-attribute disclosure will remain operative for purchase intention toward the target product but will not reliably translate into trust in the target product*.

**Figure 1:**
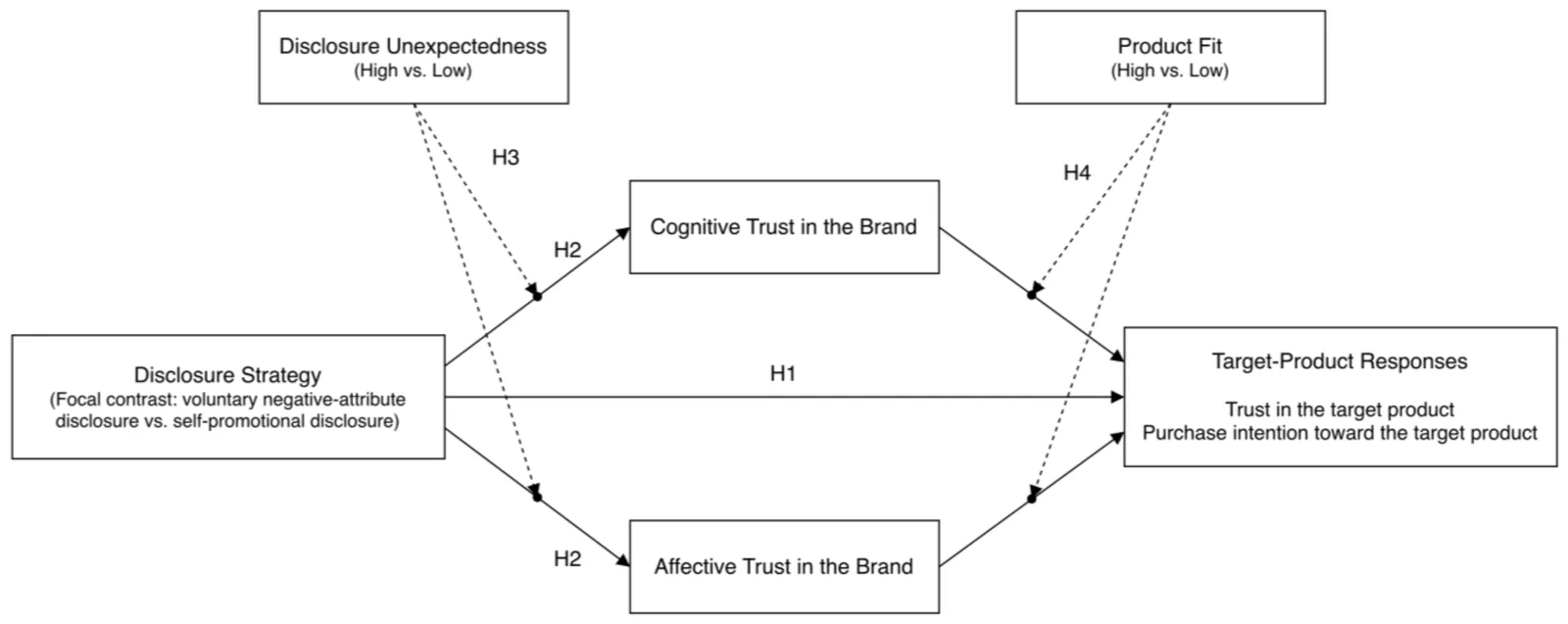
Conceptual framework of this study. Note: Solid arrows represent the focal direct and mediated relationships; dashed arrows represent the hypothesized conditional influences of unexpectedness and product fit.

Figure 1 summarizes the proposed conceptual framework.

## 3. Study 1: Establishing Cross-Product Effects and Parallel Trust Pathways

Study 1 examined whether voluntary negative-attribute disclosure concerning one product in a digital food interface influences consumers’ responses to another product offered under the same brand. It served two primary purposes. First, it tested the cross-product effects proposed in H1 by comparing voluntary negative-attribute disclosure with self-promotional disclosure and a baseline in which participants had no prior exposure to Product A. Second, it tested whether cognitive and affective trust in the brand operate as the parallel mediating pathways proposed in H2.

### 3.1. Method of Study 1

#### 3.1.1. Experimental Design and Participants

Study 1 employed a single-factor, four-condition between-subjects design. Participants were randomly assigned to a voluntary negative-attribute disclosure condition, a self-promotional disclosure condition, an objective-description condition, or a no-prior-Product-A-exposure baseline. The objective-description condition was included as an additional factual comparison condition, allowing the focal disclosure strategy to be compared with a non-evaluative presentation of Product A.

An a priori power analysis was conducted using G*Power 3.1.9.7. Assuming a medium effect size of f = 0.25, α = 0.05, and statistical power of 1 − β = 0.80, the minimum required sample size was 180. We recruited 200 participants with prior fast-food consumption experience through Credamo, a Chinese online research platform. Four responses were excluded for failing the attention check, resulting in a final sample of 196 participants. The final cell sizes were 50 in the voluntary negative-attribute disclosure condition, 49 in the self-promotional disclosure condition, 48 in the objective-description condition, and 49 in the no-prior-Product-A-exposure baseline.

Of the final sample, 43.4% were male. The largest age group was 26–35 years, accounting for 48.0% of the sample. Table 1 reports the complete demographic characteristics.

#### 3.1.2. Experimental Stimuli and Procedure

The experimental scenario involved a fictitious fast-food brand offering multiple product categories. Product A was a serving of classic French fries, and Product B was a chicken salad set positioned as a low-fat, high-fiber target product. Product A and Product B were presented sequentially rather than side by side on the same screen.

The first three conditions were mutually exclusive experimental variants constructed from a common baseline Product A interface. In all three conditions, participants viewed the same product photograph, product name, ingredient information, portion size options, quantity selector, and add-to-cart button. The experimental conditions differed only in whether an additional information module appeared above this common interface and in the content of that module.

In the voluntary negative-attribute disclosure condition, a salient red information module displayed the headline “High Sodium | High Calorie.” The accompanying text stated, “Contains 580 mg sodium (25% DV). Enjoy in moderation to avoid health burden.” Thus, the brand voluntarily foregrounded unfavorable but decision-relevant nutritional information.

In the self-promotional disclosure condition, the same interface instead included a green promotional module displaying “Premium|Crispy.” The accompanying text stated, “Premium potatoes, advanced processing to preserve nutrition. Delicious & safe.” This condition represented conventional favorable product communication.

In the objective-description condition, participants viewed the common baseline interface without an additional evaluative banner. The interface displayed the product image, name, ingredients—potatoes, vegetable oil, and salt—and the standard ordering controls but contained neither favorable promotional claims nor negative nutritional warnings. The condition therefore provided a factual, non-evaluative comparison rather than an absence-of-information condition.

In the no-prior-Product-A-exposure baseline, participants did not view the French fries interface and proceeded directly to Product B.

**Figure 2:**
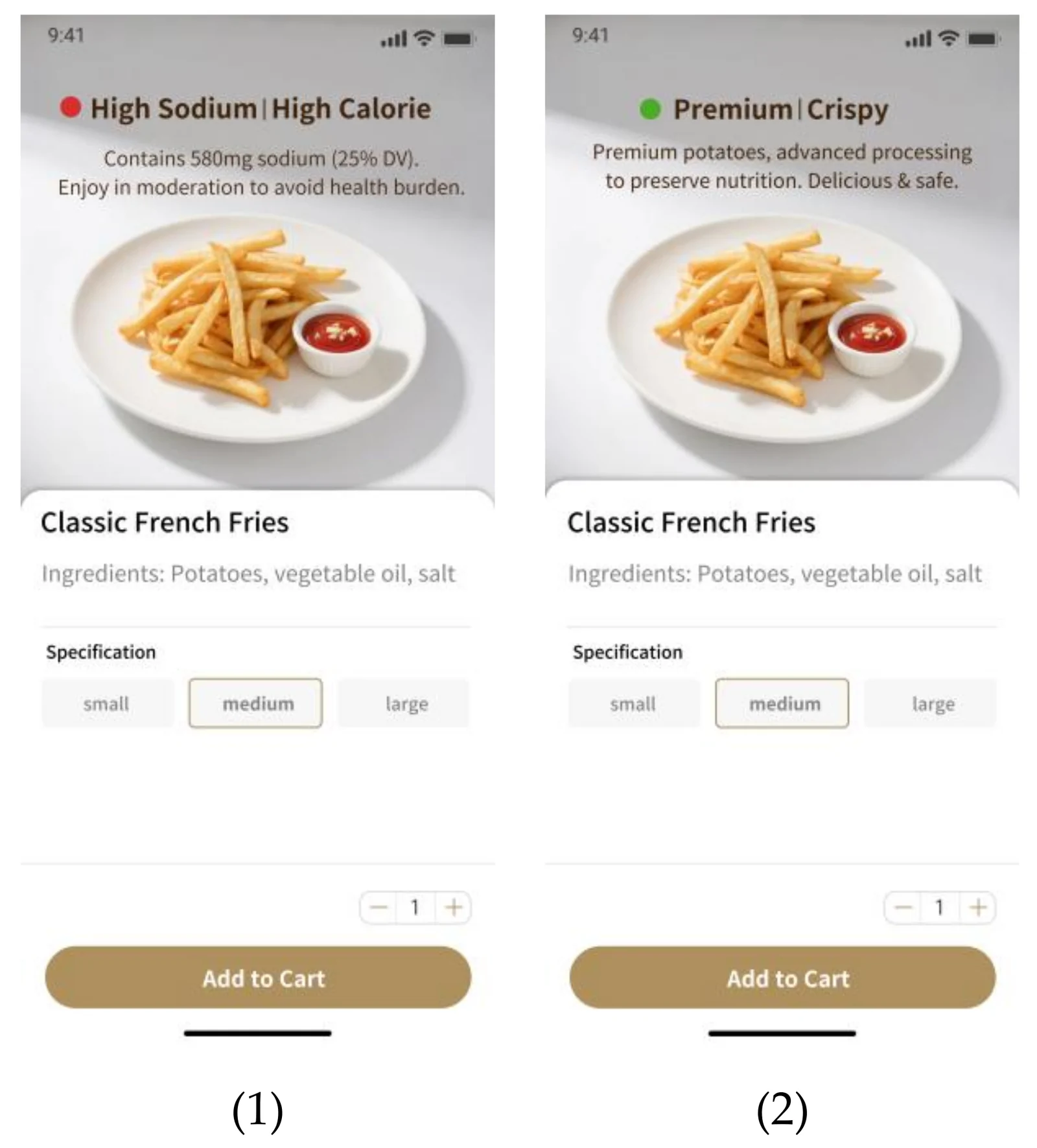

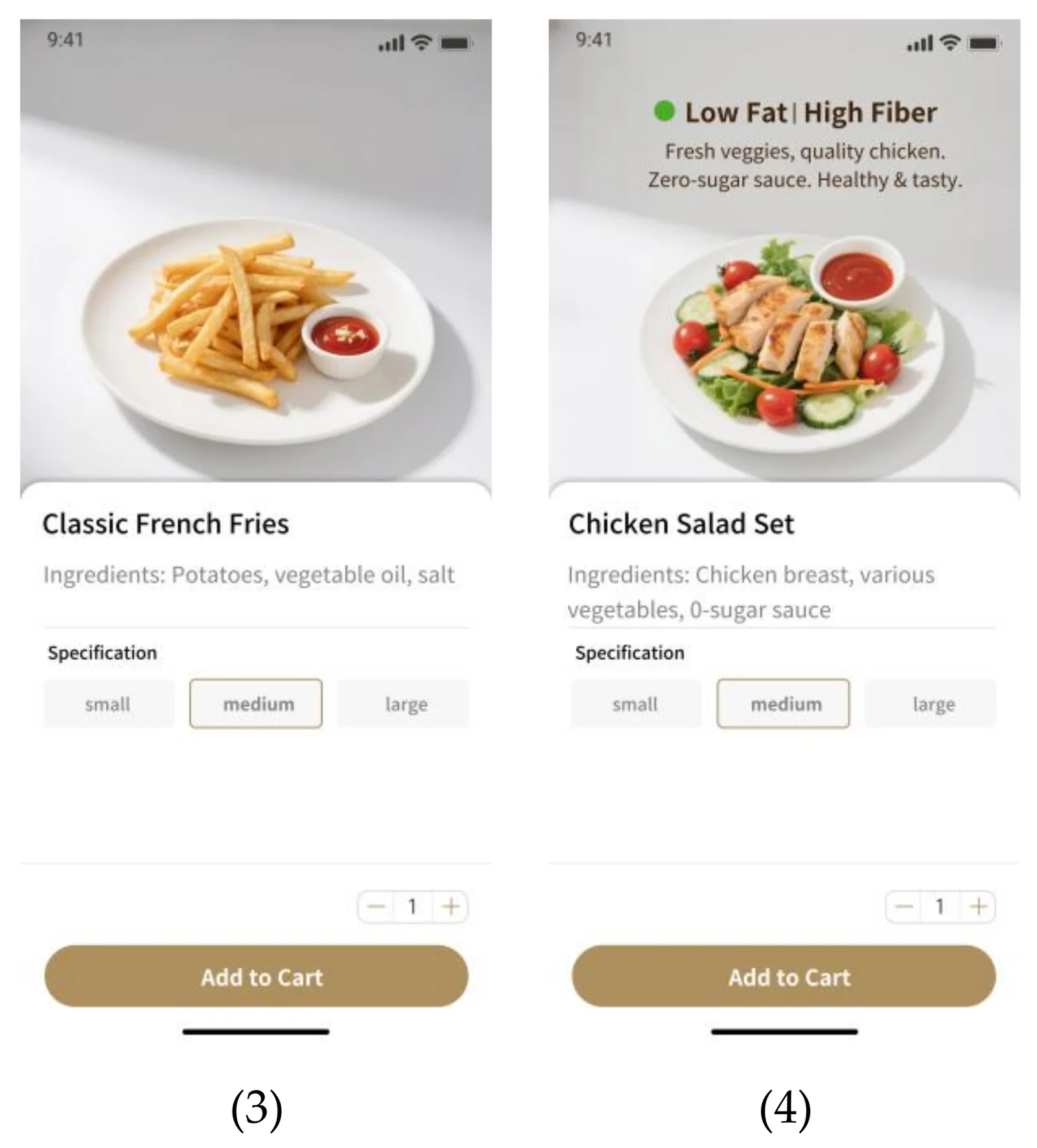
Stimuli used in Study 1. Note: Conditions 1, 2, 3, and 4 represent voluntary negative-attribute disclosure, self-promotional disclosure, objective description, and no prior Product A exposure, respectively. The Product B interface was identical across conditions.

All participants subsequently viewed the same interface for Product B, the chicken salad set. The interface displayed the headline “Low Fat | High Fiber,” together with a photograph, ingredient information, portion size options, quantity controls, and an add-to-cart button. Participants then completed the focal dependent measures, the brand trust measures, the manipulation checks, the control variables, and the demographic questions. Figure 2 presents the complete stimuli.

#### 3.1.3. Measures and Measurement Quality

All focal constructs were measured using seven-point Likert-type scales ranging from 1 (strongly disagree) to 7 (strongly agree). Cognitive trust and affective trust were each measured with four items adapted from Johnson and Grayson (2005). Cognitive trust captured beliefs concerning the brand’s reliability, honesty, and dependability, whereas affective trust captured perceived sincerity, benevolence, and concern for consumers. Target-product trust was measured with two items adapted from Chen et al. (2022), and purchase intention was measured with three items adapted from Kim et al. (2008).

The manipulation checks were study-specific direct measures developed to assess whether participants perceived the intended differences in brand transparency and Product A healthiness. Participants also reported gender, brand familiarity, and health consciousness. These three variables were included as covariates in the main effect and mediation models.

The four focal scales demonstrated acceptable to good internal consistency in Study 1. Cronbach’s alpha was 0.872 for cognitive trust, 0.866 for affective trust, 0.739 for target-product trust, and 0.723 for purchase intention.

A four-factor confirmatory factor analysis was estimated with items loading on cognitive trust, affective trust, target-product trust, and purchase intention. The model showed good fit, χ^2^ (59) = 80.55, *p* = 0.033, CFI = 0.984, TLI = 0.979, RMSEA = 0.043, 90% CI [0.013, 0.0065], SRMR = 0.034. All standardized factor loadings were significant and ranged from 0.650 to 0.835. Cognitive and affective trust were strongly correlated, r = 0.867, but a nested model constraining their latent correlation to unity fit significantly worse than the freely correlated model, Δχ^2^ (1) = 39.18, *p* < 0.001. The two trust constructs were therefore strongly associated but empirically distinguishable.

#### 3.1.4. Analytical Strategy

H1 was tested using the complete four-condition sample. Separate ordinary least squares regression models were estimated for target-product trust and purchase intention, with heteroskedasticity-robust standard errors and controls for gender, brand familiarity, and health consciousness. Planned comparisons contrasted voluntary negative-attribute disclosure with self-promotional disclosure, objective description, and no prior Product A exposure.

All parallel-mediation analyses reported below were estimated using PROCESS Model 4 with 5000 percentile bootstrap samples (Hayes, 2022). Cognitive trust and affective trust were entered simultaneously as parallel mediators, and gender, brand familiarity, and health consciousness were included as covariates.

### 3.2. Results of Study 1

#### 3.2.1. Manipulation Checks

The disclosure manipulation produced substantial differences in perceived transparency, F(3, 192) = 87.82, *p* < 0.001, R^2^ = 0.555. Perceived transparency was higher in the voluntary negative-attribute disclosure condition (M = 5.78) than in the self-promotional disclosure condition (M = 2.53), the objective-description condition (M = 2.56), and the no-prior-Product-A-exposure baseline (M = 2.16); all Bonferroni-adjusted comparisons were significant at *p* < 0.001. The self-promotional and objective-description conditions did not differ significantly.

The conditions also differed in perceived healthiness of Product A, F(3, 192) = 19.85, *p* < 0.001, R^2^= 0.217. Product A was perceived as less healthy in the voluntary negative-attribute disclosure condition (M = 2.70) than in the self-promotional disclosure condition (M = 4.73, *p* < 0.001) and the objective-description condition (M = 3.98, *p* < 0.001). These results indicated that participants noticed both the transparency-related and health-related implications of the voluntary negative-attribute disclosure.

#### 3.2.2. Main Effects and Test of H1

H1 was tested using the complete four-condition sample. Separate regression models were estimated for target-product trust and purchase intention, controlling for gender, brand familiarity, and health consciousness (see Appendix A for complete covariate coefficients).

- Target-product trust.

The regression model predicting target-product trust was significant, R^2^ = 0.175, F(6, 189) = 5.54, *p* < 0.001. The omnibus effect of disclosure condition was also significant, F(3, 189) = 3.79, *p* = 0.011.

The covariate-adjusted mean for target-product trust was 5.950 in the voluntary negative-attribute disclosure condition, compared with 5.605 in the self-promotional disclosure condition, 5.957 in the objective-description condition, and 5.672 in the no-prior-Product-A-exposure baseline. The full set of covariate-adjusted planned comparisons is presented in Table 2. As predicted, target-product trust was higher after voluntary negative-attribute disclosure than after self-promotional disclosure, B = 0.345, robust SE = 0.136, 95% CI [0.076, 0.613], *p* = 0.012, g_adj = 0.519, 95% CI [0.115, 0.923]. This comparison remained significant after Holm adjustment, *p*_Holm = 0.037. Target-product trust was also higher after voluntary negative-attribute disclosure than in the no-prior-Product-A-exposure baseline at the unadjusted planned comparison level, B = 0.278, robust SE = 0.133, 95% CI [0.015, 0.541], *p* = 0.038, g_adj = 0.418, 95% CI [0.022, 0.814]. This comparison did not remain below 0.05 in the Holm sensitivity analysis, *p*_Holm = 0.077, indicating that this contrast was less stable than the comparison with self-promotion. Voluntary negative-attribute disclosure did not differ significantly from objective description, B = −0.007, robust SE = 0.108, 95% CI [−0.220, 0.206], *p* = 0.951, g_adj = −0.010, 95% CI [−0.331, 0.311], *p*_Holm = 0.951.

- Purchase intention.

The full regression model predicting purchase intention was significant, R^2^ = 0.234, F(6, 189) = 7.89, *p* < 0.001. The omnibus effect of disclosure condition was not significant, F(3, 189) = 1.86, *p* = 0.137.

The adjusted mean for purchase intention was 5.900 in the voluntary negative-attribute disclosure condition, compared with 5.627 in the self-promotional disclosure condition, 5.916 in the objective-description condition, and 5.741 in the no-prior-Product-A-exposure baseline.

Purchase intention was directionally higher after voluntary negative-attribute disclosure than after self-promotional disclosure, but the planned contrast did not reach the conventional significance threshold, B = 0.273, robust SE = 0.154, 95% CI [−0.030, 0.575], *p* = 0.078, g_adj = 0.384, 95% CI [−0.043, 0.810]. Voluntary negative-attribute disclosure did not differ significantly from objective description, B = −0.016, robust SE = 0.117, 95% CI [−0.247, 0.215], *p* = 0.893, g_adj = −0.022, 95% CI [−0.348, 0.303], or from the no-prior-Product-A-exposure baseline, B = 0.159, robust SE = 0.139, 95% CI [−0.115, 0.433], *p* = 0.253, g_adj = 0.224, 95% CI [−0.161, 0.609].

Taken together, Study 1 supported the target-product trust component of H1 relative to self-promotion and provided less stable evidence relative to the no-prior-Product-A-exposure baseline. The purchase intention component was not supported in Study 1, although the VND–self-promotion contrast was directionally consistent with the hypothesis. H1 therefore received partial support.

#### 3.2.3. Parallel Mediation and Test of H2

The focal parallel mediation analysis compared voluntary negative-attribute disclosure (X = 1) with self-promotional disclosure (X = 0), N = 99. Voluntary negative-attribute disclosure positively predicted cognitive trust, B = 0.656, SE = 0.152, t = 4.31, *p* < 0.001, 95% CI [0.354, 0.958], and affective trust, B = 0.468, SE = 0.145, t = 3.23, *p* = 0.002, 95% CI [0.180, 0.756].

- Purchase intention.

Both mediators positively predicted purchase intention when included simultaneously. Cognitive trust was positively associated with purchase intention, B = 0.364, SE = 0.111, t = 3.27, *p* = 0.002, 95% CI [0.143, 0.585], as was affective trust, B = 0.404, SE = 0.117, t = 3.46, *p* < 0.001, 95% CI [0.172, 0.636].

The total indirect effect on purchase intention was significant, B = 0.428, BootSE = 0.145, 95% bootstrap CI [0.152, 0.728]. The specific indirect effect through cognitive trust was B = 0.239, BootSE = 0.111, 95% bootstrap CI [0.039, 0.477], and the specific indirect effect through affective trust was B = 0.189, BootSE = 0.088, 95% bootstrap CI [0.047, 0.386]. The direct effect was nonsignificant after the mediators were included, B = −0.068, SE = 0.134, *p* = 0.613, 95% CI [−0.333, 0.198]. The pattern was therefore consistent with indirect-only mediation through both trust pathways.

- Target-product trust.

Cognitive trust positively predicted target-product trust, B = 0.296, SE = 0.100, t = 2.97, *p* = 0.004, 95% CI [0.098, 0.494], and affective trust also had a positive association, B = 0.385, SE = 0.105, t = 3.68, *p* < 0.001, 95% CI [0.177, 0.592].

The total indirect effect on target-product trust was significant, B = 0.374, BootSE = 0.120, 95% bootstrap CI [0.154, 0.616]. The specific indirect effect through cognitive trust was B = 0.194, BootSE = 0.079, 95% bootstrap CI [0.044, 0.357], and the specific indirect effect through affective trust was B = 0.180, BootSE = 0.087, 95% bootstrap CI [0.045, 0.376]. The direct effect was nonsignificant, B = 0.033, SE = 0.120, *p* = 0.786, 95% CI [−0.205, 0.270]. This pattern was likewise consistent with indirect-only mediation through the two parallel trust pathways (Table 3).

The significant specific indirect effects through both cognitive and affective trust supported H2 for target-product trust and purchase intention. H2 was therefore supported.

#### 3.2.4. Exploratory Analysis of the VND–Objective-Description Contrast

Because voluntary negative-attribute disclosure and objective description produced similar downstream responses, we conducted two additional pairwise mediation analyses to determine whether the two conditions were also indistinguishable in the measured brand trust variables.

- Voluntary negative-attribute disclosure versus objective description.

The first analysis compared voluntary negative-attribute disclosure (X = 1) with objective description (X = 0), N = 98. Voluntary negative-attribute disclosure increased cognitive trust, B = 0.460, SE = 0.150, t = 3.07, *p* = 0.003, 95% CI [0.163, 0.757], but did not significantly increase affective trust, B = 0.173, SE = 0.136, *p* = 0.207, 95% CI [−0.097, 0.444].

For purchase intention, neither the total effect, B = −0.112, SE = 0.122, *p* = 0.360, 95% CI [−0.353, 0.129], nor the direct effect, B = −0.174, SE = 0.120, *p* = 0.152, was significant. The total indirect effect was also nonsignificant, B = 0.062, BootSE = 0.057, 95% bootstrap CI [−0.044, 0.181]. Neither the cognitive trust indirect effect, B = 0.004, 95% bootstrap CI [−0.090, 0.105], nor the affective trust indirect effect, B = 0.058, 95% bootstrap CI [−0.030, 0.178], was significant.

For target-product trust, neither the total effect, B = −0.056, SE = 0.113, *p* = 0.622, 95% CI [−0.280, 0.168], nor the direct effect, B = −0.160, SE = 0.110, *p* = 0.150, was significant. The aggregate indirect effect was positive, B = 0.104, BootSE = 0.057, 95% bootstrap CI [0.001, 0.224], but neither specific indirect effect was individually significant: cognitive trust, B = 0.071, 95% bootstrap CI [−0.017, 0.200], and affective trust, B = 0.033, 95% bootstrap CI [−0.028, 0.127].

The clearest difference between voluntary negative-attribute disclosure and objective description therefore occurred in the proximal cognitive trust judgment. This difference did not translate into a significant specific indirect effect or a downstream advantage on either outcome.

- Objective description versus self-promotion.

The second analysis compared objective description (X = 1) with self-promotion (X = 0), N = 97. Objective description did not significantly increase cognitive trust, B = 0.213, SE = 0.192, *p* = 0.269, or affective trust, B = 0.300, SE = 0.179, *p* = 0.097.

Objective description produced a positive total effect on purchase intention, B = 0.451, SE = 0.160, *p* = 0.006, 95% CI [0.134, 0.768], and a positive direct effect after the mediators were included, B = 0.289, SE = 0.128, *p* = 0.026, 95% CI [0.036, 0.543]. The total indirect effect was nonsignificant, B = 0.162, BootSE = 0.118, 95% bootstrap CI [−0.030, 0.428]. Neither the cognitive trust indirect effect, B = 0.026, 95% bootstrap CI [−0.031, 0.157], nor the affective trust indirect effect, B = 0.137, 95% bootstrap CI [−0.022, 0.346], was significant.

A similar pattern emerged for target-product trust. Objective description produced a significant total effect, B = 0.456, SE = 0.147, *p* = 0.003, 95% CI [0.163, 0.748], and a significant direct effect, B = 0.346, SE = 0.125, *p* = 0.007, 95% CI [0.098, 0.595]. However, the total indirect effect was nonsignificant, B = 0.109, BootSE = 0.102, 95% bootstrap CI [−0.056, 0.343], and neither specific indirect effect excluded zero.

Taken together, these analyses show that the absence of downstream differences did not make voluntary negative-attribute disclosure and objective description empirically interchangeable. VND generated significantly higher cognitive trust than objective description, whereas the downstream advantage of objective description over self-promotion was not transmitted through either measured trust pathway. Collapsing the two conditions would therefore obscure a meaningful difference in cognitive trust formation. Because the present research focuses on VND, the specific process underlying the objective-description condition is not pursued further here.

### 3.3. Discussion of Study 1

Study 1 provides initial evidence that a disclosure decision concerning Product A can influence consumers’ responses to Product B under the same brand. Relative to self-promotional disclosure, voluntary negative-attribute disclosure increased target-product trust, and this contrast remained significant in the multiplicity-adjusted sensitivity analysis. Target-product trust was also higher than in the no-prior-Product-A-exposure baseline at the unadjusted planned comparison level, although this comparison was more sensitive to adjustment. Purchase intention followed the predicted direction but did not differ significantly across the focal comparisons. Study 1 therefore provided clearer support for the target-product trust component of H1 than for its purchase intention component, resulting in partial support for H1.

The focal mediation analysis further showed that voluntary negative-attribute disclosure increased both cognitive and affective trust relative to self-promotion. Each trust dimension carried a significant indirect effect on target-product trust and purchase intention, while the residual direct effects were nonsignificant. These results support the proposed parallel trust pathways and are consistent with indirect-only mediation. H2 was therefore supported.

The additional analyses also clarify why the objective-description condition should not be treated as interchangeable with VND. Although the two conditions produced similar downstream responses, VND generated a stronger cognitive trust response. By contrast, the downstream advantage of objective description over self-promotion was not transmitted through either measured trust pathway. Thus, a common downstream pattern did not imply an identical brand trust process. The incremental cognitive trust response preserves the theoretical relevance of VND as the focal disclosure strategy, even though Study 1 does not establish that negative content is necessary for favorable downstream responses.

The divergence between target-product trust and purchase intention is theoretically informative. Target-product trust is a relatively proximal evaluation of whether Product B appears credible and dependable, whereas purchase intention is a more distal and integrative response that also depends on product preferences, current needs, price, available alternatives, and perceived behavioral feasibility (Ajzen, 1991; Kim et al., 2008). A disclosure-based brand inference may therefore translate more readily into confidence in the target product than into an immediate intention to purchase it. The significant indirect effects on purchase intention indicate that cognitive and affective trust can contribute to this more distal response. Next, Study 2 examines whether the unexpectedness of the disclosed drawback changes the persistence of these two trust pathways.

## 4. Study 2: Unexpectedness and Trust Pathway Persistence

Study 2 examined how the unexpectedness of a voluntarily disclosed negative attribute changes the formation of cognitive and affective trust and their subsequent transmission to target-product trust and purchase intention. Building on Study 1, the study retained voluntary negative-attribute disclosure as the focal disclosure strategy and introduced a low- versus high-unexpectedness manipulation. It tested H3 by examining whether high unexpectedness attenuates the cognitive trust pathway while the affective trust pathway remains operative.

### 4.1. Method of Study 2

#### 4.1.1. Pretest for Study 2

An independent pretest (N = 100) assessed whether French fries and tomato beef soup represented the intended low- and high-unexpectedness contexts. Participants were randomly assigned to view an unbranded image of either tomato beef soup or French fries (n = 50 per condition). They first evaluated the product’s perceived healthiness. They were then asked to imagine learning that the product contained 620 mg of sodium per serving and evaluated how unexpected this information was and how inconsistent it was with their usual impression of the food.

Independent-samples *t*-tests showed that tomato beef soup was perceived as healthier (M = 5.20, SD = 0.90) than French fries (M = 3.02, SD = 0.89), t(98) = 12.14, *p* < 0.001. The high-sodium information was also perceived as more unexpected for tomato beef soup (M = 5.14, SD = 1.14) than for French fries (M = 2.94, SD = 1.25), t(98) = 9.18, *p* < 0.001. Similarly, the information was rated as more inconsistent with participants’ usual impressions of tomato beef soup (M = 5.10, SD = 0.89) than French fries (M = 2.86, SD = 1.01), t(98) = 11.78, *p* < 0.001. These results supported the intended healthiness and unexpectedness contrast between the two Product A categories.

#### 4.1.2. Experimental Design and Participants

Study 2 employed a 4 (disclosure strategy: voluntary negative-attribute disclosure vs. self-promotional disclosure vs. objective description vs. no prior Product A exposure) × 2 (unexpectedness: low vs. high) between-subjects design. Participants were randomly assigned to one of the eight experimental conditions.

A total of 440 participants with prior fast-food consumption experience were recruited through Credamo, a Chinese online research platform. Six participants who failed the attention check were excluded, resulting in a final sample of 434 participants. Of the final sample, 44.5% were male, and the largest age group was 26–35 years, accounting for 46.5% of the sample. Table 4 reports the complete demographic characteristics.

#### 4.1.3. Experimental Stimuli and Procedure

Study 2 followed the same general interface design, disclosure strategy manipulations, and procedure as Study 1. The low-unexpectedness condition used the French fries stimulus from Study 1, whereas the high-unexpectedness condition replaced Product A with tomato beef soup. In the latter condition, the voluntary negative-attribute disclosure module displayed “High Sodium|High Calorie” and stated, “Contains 620 mg sodium (31% DV). Enjoy in moderation to avoid health burden.” The corresponding self-promotional condition presented a favorable product quality message, whereas the objective-description condition retained the factual product information without an additional evaluative module.

**Figure 3:**
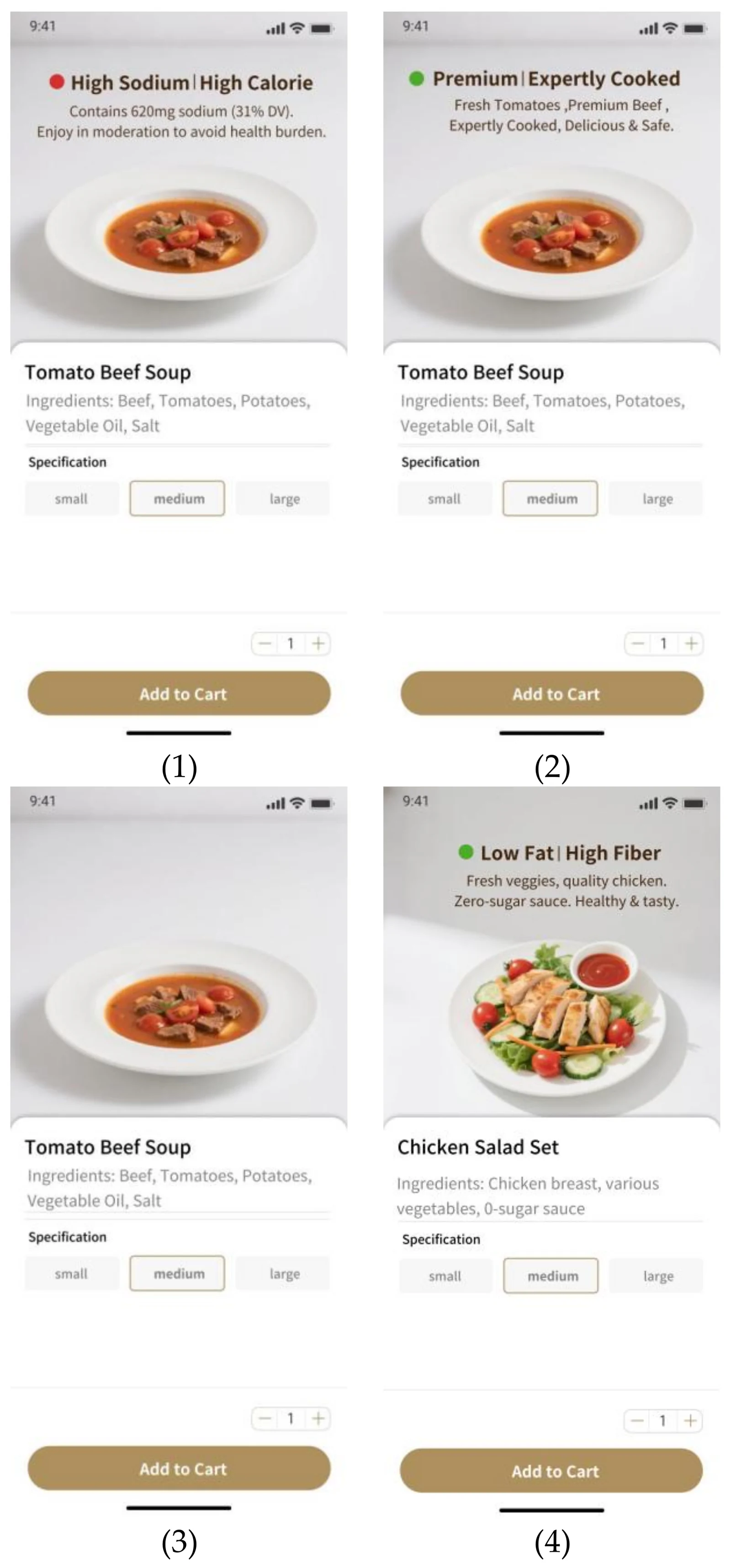
Stimuli used in Study 2. Note: Panels (1)–(4) show the high-unexpectedness voluntary negative-attribute disclosure condition, the high-unexpectedness self-promotional disclosure condition, the high-unexpectedness objective-description condition, and the common Product B interface, respectively. The low-unexpectedness condition used French fries, whereas the high-unexpectedness condition used tomato beef soup. Within each Product A category, the three disclosure strategy conditions shared a common page structure. Participants in the no-prior-Product-A-exposure condition proceeded directly to the common Product B interface.

Across conditions, the Product A pages shared the same basic ordering interface structure. Participants in the first three disclosure conditions viewed Product A according to their assigned disclosure strategy and unexpectedness condition, whereas those in the no-prior-Product-A-exposure condition proceeded directly to Product B. All participants then viewed the same chicken salad set interface used in Study 1 and completed the target-product measures, brand trust measures, manipulation checks, control variables, and demographic questions. Figure 3 presents the Study 2 stimuli.

#### 4.1.4. Measures and Analytical Strategy

Study 2 used the same seven-point measures and manipulation checks as Study 1. Gender, brand familiarity, and health consciousness were included as covariates. Cronbach’s alpha was 0.803 for cognitive trust, 0.799 for affective trust, 0.747 for target-product trust, and 0.736 for purchase intention.

H1 was examined using the complete four-condition sample. Separate ordinary least squares regression models were estimated for target-product trust and purchase intention, with heteroskedasticity-robust standard errors and controls for gender, brand familiarity, and health consciousness. Planned comparisons contrasted voluntary negative-attribute disclosure with self-promotional disclosure, objective description, and no prior Product A exposure. Effect sizes were summarized using covariate-adjusted standardized contrasts, and Holm adjustment was used as a multiplicity sensitivity analysis.

H3 was tested using PROCESS Model 7 with 5000 percentile bootstrap samples (Hayes, 2022) in the voluntary negative-attribute disclosure and self-promotional disclosure subsample (N = 218). Voluntary negative-attribute disclosure was coded 1, and self-promotion was coded 0; low unexpectedness was coded 0, and high unexpectedness was coded 1. Cognitive trust and affective trust were modeled as parallel mediators, with unexpectedness moderating both first-stage paths. Separate models were estimated for target-product trust and purchase intention, controlling for gender, brand familiarity, and health consciousness. Conditional indirect effects and indices of moderated mediation were evaluated using 95% bootstrap confidence intervals.

### 4.2. Results of Study 2

#### 4.2.1. Manipulation Checks

The disclosure conditions differed substantially in perceived transparency, F(3, 430) = 183.14, *p* < 0.001, R^2^ = 0.550. Perceived transparency was higher in the voluntary negative-attribute disclosure condition (M = 5.61) than in the self-promotional disclosure condition (M = 2.31), the objective-description condition (M = 2.55), and the no-prior-Product-A-exposure condition (M = 2.15); all Bonferroni-adjusted comparisons were significant at *p* < 0.001. The self-promotional and objective-description conditions did not differ significantly.

The conditions also differed in the perceived healthiness of Product A, F(3, 430) = 40.74, *p* < 0.001, R^2^ = 0.202. Product A was perceived as less healthy in the voluntary negative-attribute disclosure condition (M = 3.25) than in the self-promotional disclosure condition (M = 5.22, *p* < 0.001) and the objective-description condition (M = 4.79, *p* < 0.001). These results indicated that participants noticed both the transparency-related and health-related implications of the voluntary negative-attribute disclosure.

#### 4.2.2. Main Effects and Additional Evidence for H1

- Target-product trust.

The regression model predicting target-product trust was significant, R^2^ = 0.109, F(6, 427) = 8.48, *p* < 0.001.

The covariate-adjusted mean for target-product trust was 5.926 in the voluntary negative-attribute disclosure condition, compared with 5.699 in the self-promotional disclosure condition, 5.878 in the objective-description condition, and 5.701 in the no-prior-Product-A-exposure condition. The full set of covariate-adjusted planned comparisons is presented in Table 5. Target-product trust was higher after voluntary negative-attribute disclosure than after self-promotional disclosure, B = 0.227, robust SE = 0.089, 95% CI [0.053, 0.401], *p* = 0.011. This contrast remained significant after Holm adjustment, *p*_Holm = 0.032. Target-product trust was also higher after voluntary negative-attribute disclosure than in the no-prior-Product-A-exposure condition, B = 0.225, robust SE = 0.090, 95% CI [0.049, 0.401], *p* = 0.012. This contrast likewise remained significant after Holm adjustment, *p*_Holm = 0.032. Voluntary negative-attribute disclosure did not differ significantly from objective description, B = 0.049, robust SE = 0.082, 95% CI [−0.112, 0.209], *p* = 0.552.

- Purchase intention.

The regression model predicting purchase intention was significant, R^2^ = 0.193, F(6, 427) = 16.69, *p* < 0.001. The covariate-adjusted mean for purchase intention was 5.826 in the voluntary negative-attribute disclosure condition, compared with 5.612 in the self-promotional disclosure condition, 5.873 in the objective-description condition, and 5.750 in the no-prior-Product-A-exposure condition.

Purchase intention was higher after voluntary negative-attribute disclosure than after self-promotional disclosure at the unadjusted planned comparison level, B = 0.213, robust SE = 0.098, 95% CI [0.021, 0.406], *p* = 0.030. This contrast did not remain significant after Holm adjustment, *p*_Holm = 0.090.

Voluntary negative-attribute disclosure did not differ significantly from objective description, B = −0.048, robust SE = 0.083, 95% CI [−0.211, 0.116], *p* = 0.568, or from the no-prior-Product-A-exposure condition, B = 0.076, robust SE = 0.094, 95% CI [−0.109, 0.262], *p* = 0.420.

Taken together, Study 2 provided additional but partial support for H1. Voluntary negative-attribute disclosure increased target-product trust relative to both self-promotion and no prior Product-A exposure, and both contrasts remained significant after Holm adjustment. For purchase intention, the VND–self-promotion contrast was significant at the unadjusted level but was more sensitive to multiplicity adjustment. The evidence for H1 was therefore stronger and more stable for target-product trust than for purchase intention.

#### 4.2.3. Moderated Mediation and Test of H3

Unexpectedness significantly moderated the effects of disclosure strategy on both cognitive and affective trust. For cognitive trust, the interaction between disclosure strategy and unexpectedness was negative and significant, B = −0.541, SE = 0.225, t = −2.41, *p* = 0.017, 95% CI [−0.984, −0.098]. Voluntary negative-attribute disclosure increased cognitive trust under low unexpectedness, B = 0.636, SE = 0.165, *p* < 0.001, 95% CI [0.310, 0.962], but not under high unexpectedness, B = 0.095, SE = 0.151, *p* = 0.529, 95% CI [−0.202, 0.392].

The interaction predicting affective trust was also negative and significant, B = −0.386, SE = 0.179, t = −2.16, *p* = 0.032, 95% CI [−0.738, −0.034]. Voluntary negative-attribute disclosure increased affective trust under low unexpectedness, B = 0.620, SE = 0.132, *p* < 0.001, 95% CI [0.361, 0.880]. Under high unexpectedness, this effect was smaller and did not reach the conventional significance threshold, B = 0.234, SE = 0.120, *p* = 0.052, 95% CI [−0.002, 0.471].

For purchase intention, the cognitive trust indirect effect was significant under low unexpectedness but not under high unexpectedness. The affective trust indirect effect, by contrast, remained significant under both conditions. The indices of moderated mediation for both pathways were negative, and neither bootstrap confidence interval included zero.

The same pattern emerged for target-product trust. The cognitive trust indirect effect was significant under low unexpectedness but not under high unexpectedness, whereas the affective trust indirect effect remained significant at both levels. Both corresponding indices of moderated mediation were negative and excluded zero. Table 6 reports the conditional indirect effects and moderated mediation indices for the two outcomes.

Taken together, high unexpectedness attenuated both trust pathways. However, the cognitive pathway was no longer operative under high unexpectedness, whereas the affective pathway continued to transmit the effect of voluntary negative-attribute disclosure to both target-product outcomes. This pattern of cognitive attenuation and affective persistence supported H3.

### 4.3. Discussion of Study 2

Study 2 reinforced the evidence that voluntary negative-attribute disclosure can influence responses beyond the disclosed product. Relative to self-promotion and no prior Product A exposure, voluntary negative-attribute disclosure produced higher target-product trust, and both contrasts remained significant after Holm adjustment. The purchase intention advantage over self-promotion was significant in the planned comparison but did not remain significant after adjustment. Study 2 therefore provided additional partial support for H1, with more stable evidence for target-product trust than for purchase intention.

More importantly, unexpectedness shaped the formation and transmission of brand trust. High unexpectedness weakened the effects of voluntary negative-attribute disclosure on both cognitive and affective trust. The cognitive trust indirect effects were significant under low unexpectedness but not under high unexpectedness, whereas the affective trust indirect effects remained significant under both conditions. The moderated mediation indices further showed that both pathways were attenuated as unexpectedness increased. Taken together, these findings reveal a pattern of cognitive attenuation and affective persistence, supporting H3.

One plausible interpretation is that expectancy-inconsistent negative information heightens attention to the disclosed drawback and makes it more difficult to integrate the disclosure into a stable judgment of brand reliability. This may constrain cognitive trust, which rests on assessments of consistency, honesty, and dependability. At the same time, the decision to disclose unfavorable information may retain relational meaning concerning sincerity and concern for informed consumer choice, allowing affective trust to continue transmitting the disclosure effect even as that pathway becomes weaker under high unexpectedness (Folkes, 1984; Friestad & Wright, 1994; Meyers-Levy & Tybout, 1989).

Study 2 thus identifies unexpectedness as a boundary condition for the formation of disclosure-based brand trust: cognitive and affective pathways do not remain equally operative when a disclosed drawback violates category expectations. Study 3 shifts from this trust-formation boundary to a trust-translation boundary by examining whether brand-level cognitive and affective trust continue to support target-product trust and purchase intention when the disclosed and target products have low product fit.

## 5. Study 3: Product Fit and Trust Translation Across Product Boundaries

Study 2 identified unexpectedness as a boundary condition on the formation of disclosure-based brand trust. Study 3 shifted attention to a subsequent question: whether brand-level trust continues to support responses to another product when the disclosed and target products are low in fit. Specifically, Study 3 tested the conditional pattern predicted in H4—whether, under low product fit, cognitive and affective trust pathways remain operative for purchase intention but do not reliably translate into target-product trust.

Product fit was manipulated by presenting either a corn cup or a branded cotton towel as Product B after participants viewed the disclosure concerning Product A. The corn cup represented a related food product, whereas the towel represented a product portfolio extension outside the food category. This contrast allowed target-product trust and purchase intention to be examined separately when the category relationship between the disclosed and target products was either relatively strong or weak.

### 5.1. Method of Study 3

#### 5.1.1. Pretest for Study 3

An independent pretest (N = 60; 56.7% female) was conducted to select the target products for the product fit manipulation. Participants first viewed French fries as the focal fast-food product and then rated the perceived similarity between this product and eight candidate target products.

The corn cup was perceived as substantially higher in product fit (M = 5.88, SD = 1.08) than the towel (M = 1.71, SD = 0.77), t(59) = 25.43, *p* < 0.001. The main experiment therefore used a “Lightly Sweet | Low-Calorie” corn cup as the high-fit target product and a “Soft Skin | Breathable” branded cotton towel as the low-fit target product.

#### 5.1.2. Experimental Design and Participants

Study 3 employed a 3 (disclosure strategy: voluntary negative-attribute disclosure vs. self-promotional disclosure vs. objective description) × 2 (product fit: low vs. high) between-subjects design. Participants were randomly assigned to one of the six experimental conditions.

A total of 190 participants with prior fast-food consumption experience were recruited through Credamo, a Chinese online research platform. Two participants who failed the attention check were excluded, resulting in a final sample of 188 participants. Of the final sample, 48.4% were male, and the largest age group was 26–35 years, accounting for 54.3% of the sample. Table 7 reports the complete demographic characteristics.

#### 5.1.3. Experimental Stimuli and Procedure

Study 3 used the same French fries Product A interface and disclosure strategy manipulations as Study 1. Participants viewed Product A under the voluntary negative-attribute disclosure, self-promotional disclosure, or objective-description condition. The general interface structure and disclosure modules were unchanged from Study 1.

Product fit was manipulated through Product B. In the high-fit condition, participants viewed a corn cup interface displaying the headline “Lightly Sweet | Low-Calorie.” In the low-fit condition, participants viewed a branded cotton towel interface displaying “Soft Skin | Breathable.” The two Product B pages followed the same mobile ordering interface format and differed in product category, image, product information, and the accompanying category-appropriate promotional description.

**Figure 4:**
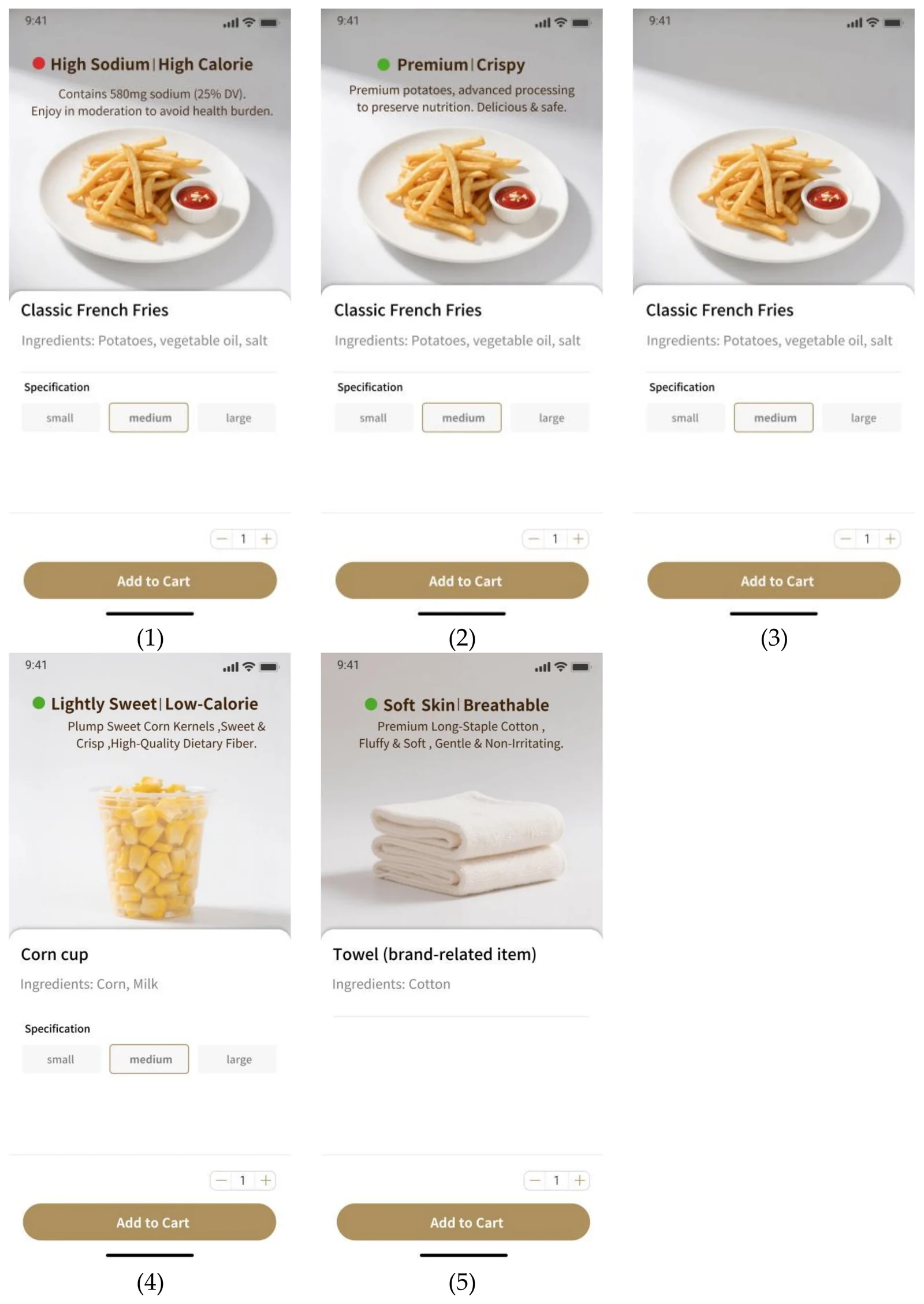
Stimuli used in Study 3. Note: Panels (1)–(5) show the voluntary negative-attribute disclosure condition for Product A, the self-promotional disclosure condition for Product A, the objective-description condition for Product A, the high-fit Product B condition (corn cup), and the low-fit Product B condition (branded cotton towel), respectively. Product A used the French fries interfaces from Study 1 under the voluntary negative-attribute disclosure, self-promotional disclosure, and objective-description conditions. Product B was either a corn cup in the high-fit condition or a branded cotton towel in the low-fit condition. The two Product B pages used the same general mobile ordering interface format.

Participants first read instructions describing a market research survey and then viewed Product A according to their assigned disclosure condition. They subsequently viewed either the high-fit or low-fit Product B interface and completed the target-product measures, brand trust measures, manipulation checks, control variables, and demographic questions. Figure 4 presents the Study 3 stimuli.

#### 5.1.4. Measures and Analytical Strategy

Study 3 used the same seven-point focal measures and disclosure manipulation checks as Study 1. Perceived product fit served as the manipulation check for the fit condition. Gender, brand familiarity, and health consciousness were included as covariates. Cronbach’s alpha was 0.900 for cognitive trust, 0.889 for affective trust, 0.829 for target-product trust, and 0.808 for purchase intention.

H4 was tested using PROCESS Model 14 with 5000 percentile bootstrap samples (Hayes, 2022). Consistent with the focal comparison specified in H4, the analysis included participants in the voluntary negative-attribute disclosure and self-promotional disclosure conditions (N = 127). Voluntary negative-attribute disclosure was coded 1, and self-promotion was coded 0; low product fit was coded 0, and high product fit was coded 1. The experimentally assigned fit condition, rather than the post-exposure manipulation check rating, was entered as the moderator.

Cognitive trust and affective trust were modeled as parallel mediators, with product fit moderating both second-stage paths from the mediators to the outcome. Separate models were estimated for target-product trust and purchase intention, controlling for gender, brand familiarity, and health consciousness. H4 concerned the configuration of conditional indirect effects under low product fit; indices of moderated mediation were examined separately to assess whether indirect effect magnitudes differed across the two fit conditions.

### 5.2. Results of Study 3

#### 5.2.1. Manipulation Checks

Disclosure strategy significantly affected perceived transparency, F(2, 185) = 26.05, *p* < 0.001. Perceived transparency was higher in the voluntary negative-attribute disclosure condition than in both the self-promotional and objective-description conditions, with both comparisons significant at *p* < 0.001. The self-promotional and objective-description conditions did not differ significantly.

Disclosure strategy also affected the perceived healthiness of Product A, F(2, 185) = 6.22, *p* = 0.002. Perceived healthiness was higher in the self-promotional condition than in the voluntary negative-attribute disclosure and objective-description conditions.

The product fit manipulation was also effective. Perceived fit was lower in the low-fit condition (M = 2.23, SD = 1.34) than in the high-fit condition (M = 5.68, SD = 1.07), t(186) = −19.50, *p* < 0.001. These results supported the intended disclosure strategy and product fit manipulations.

#### 5.2.2. Conditional Indirect Effects Across Product Fit and Test of H4

Voluntary negative-attribute disclosure significantly increased both mediators relative to self-promotion. Its effect was significant for cognitive trust, B = 0.627, SE = 0.158, t = 3.98, *p* < 0.001, 95% CI [0.315, 0.939], and for affective trust, B = 0.512, SE = 0.147, t = 3.48, *p* = 0.001, 95% CI [0.221, 0.803].

None of the four second-stage interactions between brand trust and product fit reached the conventional significance threshold. For purchase intention, the cognitive trust × product fit interaction was not significant, B = −0.123, SE = 0.234, t = −0.53, *p* = 0.600, 95% CI [−0.586, 0.340], nor was the affective trust × product fit interaction, B = −0.255, SE = 0.246, t = −1.04, *p* = 0.302, 95% CI [−0.741, 0.232]. For target-product trust, neither the cognitive trust × product fit interaction, B = 0.111, SE = 0.264, t = 0.42, *p* = 0.673, 95% CI [−0.411, 0.633], nor the affective trust × product fit interaction, B = 0.541, SE = 0.277, t = 1.95, *p* = 0.053, 95% CI [−0.008, 1.090], was significant.

Under low product fit, the indirect effect on purchase intention was significant through both cognitive trust, indirect effect = 0.337, 95% bootstrap CI [0.056, 0.721], and affective trust, indirect effect = 0.283, 95% bootstrap CI [0.051, 0.565]. By contrast, neither pathway significantly transmitted the disclosure effect to target-product trust under low fit: the cognitive trust indirect effect was 0.200, 95% bootstrap CI [−0.124, 0.603], and the affective trust indirect effect was −0.101, 95% bootstrap CI [−0.434, 0.223].

Under high product fit, all four conditional indirect effects were significant. Cognitive and affective trust transmitted the disclosure effect to purchase intention, with indirect effects of 0.260, 95% bootstrap CI [0.072, 0.490], and 0.153, 95% bootstrap CI [0.001, 0.415], respectively. They also transmitted the effect to target-product trust, with indirect effects of 0.270, 95% bootstrap CI [0.082, 0.508], and 0.176, 95% bootstrap CI [0.013, 0.468], respectively.

However, none of the four indices of moderated mediation excluded zero. The predicted low-fit configuration was therefore observed: under low product fit, both trust pathways remained operative for purchase intention but neither reliably translated into target-product trust. This conditional pattern supported H4. At the same time, the nonsignificant indices did not establish that the magnitudes of the indirect effects differed statistically between the low- and high-fit conditions. Table 8 presents the complete results.

### 5.3. Discussion of Study 3

Study 3 examined how disclosure-generated brand trust translates into target-product responses when the disclosed and target products are weakly related. Under low product fit, voluntary negative-attribute disclosure influenced purchase intention through both cognitive and affective trust, whereas neither pathway reliably transmitted the effect to target-product trust. This predicted conditional configuration supported H4. The findings therefore indicate that the downstream implications of brand trust may diverge across target-product trust and purchase intention when the target product lies outside the disclosed product’s category.

This divergence is consistent with the different inferential requirements of the two outcomes. Target-product trust is a relatively proximal judgment requiring product-specific confidence that the target product is reliable and credible. When the disclosed food product and the target lifestyle product are weakly related, the shared brand may not provide sufficient category-relevant evidence for extending confidence from one product to the other (Aaker & Keller, 1990; Roehm & Tybout, 2006). Purchase intention is a more distal and integrative response that can draw not only on confidence in the target product but also on broader brand orientation, perceived value, preferences, needs, situational goals, and behavioral feasibility (Ajzen, 1991; Kim et al., 2008; Lim et al., 2006). Cognitive trust may therefore support a general willingness to transact with a brand perceived as reliable, while affective trust may support willingness to choose a brand perceived as sincere and consumer-oriented, even when neither form of brand trust establishes strong product-specific confidence.

The results nevertheless require a clear inferential boundary. None of the four second-stage interactions reached the conventional significance threshold, and all four indices of moderated mediation included zero. Although both trust pathways significantly transmitted the disclosure effect to both outcomes under high product fit, the analyses did not establish statistically significant differences in indirect effect magnitudes between the low- and high-fit conditions. The findings should therefore be interpreted as a theoretically predicted conditional pattern under low fit rather than as definitive evidence that product fit significantly moderated the indirect effects.

Study 3 extends the preceding studies by distinguishing the formation of brand trust from its translation to specific downstream responses. Studies 1 and 2 examined whether disclosure-generated cognitive and affective trust arise and remain operative under different disclosure conditions. Study 3 indicates that once formed, brand trust need not translate uniformly into product-specific confidence and behavioral intention when the target product is category-distant. Thus, cross-product trust transfer depends not only on whether consumers form trust in the brand but also on the inferential requirements of the particular target-product response.

## 6. General Discussion

### 6.1. Summary of Findings

Across three online experiments, this research examined whether a product-level disclosure choice in a digital food interface can acquire brand-level meaning and influence consumers’ responses to another product under the same brand. Studies 1 and 2 provided convergent but partial support for H1. In both studies, voluntary negative-attribute disclosure produced higher target-product trust than self-promotional disclosure, and these contrasts remained significant after Holm adjustment. Relative to no prior Product A exposure, the target-product trust advantage was significant but adjustment-sensitive in Study 1 and remained significant after Holm adjustment in Study 2. The evidence for purchase intention was less stable: although voluntary negative-attribute disclosure produced a favorable contrast relative to self-promotion at the unadjusted planned comparison level, this contrast did not remain significant after Holm adjustment. The cross-product effect was therefore more consistently expressed in target-product trust than in purchase intention.

Study 1 also clarified the process underlying the cross-product pattern in H1 and supported H2. Relative to self-promotion, voluntary negative-attribute disclosure increased both cognitive and affective trust, and each form of brand trust carried significant indirect effects on target-product trust and purchase intention. Although voluntary negative-attribute disclosure and objective description produced statistically comparable downstream outcomes, their effects on brand trust formation differed. Voluntary negative-attribute disclosure generated significantly higher cognitive trust than objective description. Moreover, when each condition was compared with self-promotion, only voluntary negative-attribute disclosure produced significant increases in both cognitive and affective trust. The similarity in downstream outcomes therefore coexisted with a clear difference in brand trust formation, highlighting the distinctive capacity of voluntary negative-attribute disclosure to build brand-level trust, especially cognitive trust.

Study 2 identified unexpectedness as a boundary condition on the formation of disclosure-based brand trust. High unexpectedness attenuated the effects of voluntary negative-attribute disclosure on both cognitive and affective trust. The cognitive trust indirect effects were significant under low unexpectedness but not under high unexpectedness, whereas the affective trust indirect effects remained significant under both conditions. The significant negative indices of moderated mediation showed that both pathways became weaker as unexpectedness increased. This pattern supported H3 as one of cognitive attenuation and affective persistence.

Study 3 shifted from the formation of brand trust to its translation into distinct target-product responses. Under low product fit, both cognitive and affective trust transmitted the disclosure effect to purchase intention, whereas neither pathway reliably transmitted the effect to target-product trust. This predicted conditional configuration supported H4. Under high fit, both trust pathways were significant for both outcomes. However, the second-stage interactions and indices of moderated mediation did not establish statistically significant differences in indirect effect magnitudes between the low- and high-fit conditions. Taken together, the three studies show that voluntary negative-attribute disclosure can inform brand-level trust, while the formation and downstream translation of that trust vary with the unexpectedness of the disclosed attribute, the relationship between products, and the inferential requirements of the outcome being considered.

### 6.2. Theoretical Implications

This research offers five theoretical implications for research on product-level transparency, digital consumer interfaces, brand trust, and cross-product transfer.

First, the research extends the study of voluntary product disclosure from focal-product responses to cross-product consequences. Prior research showed that voluntarily disclosing unhealthy product attributes in a digital food-ordering context can increase perceived transparency and trust toward the disclosed product (Sun et al., 2025). The present research examines the next stage of that process: whether a disclosure concerning Product A changes consumers’ view of the brand and consequently affects Product B. In doing so, it connects product-level transparency with brand extension and spillover research, which has shown that associations attached to one branded offering can affect evaluations of another offering when consumers recognize a meaningful connection through the shared brand (Aaker & Keller, 1990; C. Peng et al., 2023; Roehm & Tybout, 2006). The contribution is therefore not simply that disclosure affects product evaluation, but that a communication choice concerning one product can become a brand-level inference with consequences elsewhere in the portfolio.

Second, the research clarifies the theoretical role of digital food interfaces. Digital interfaces are not merely neutral channels in which the same fixed product information is reproduced. Their layered information capacity, relatively low updating and replication costs, integration with the point of choice, and portfolio-linked navigation make product-level disclosure easier to implement repeatedly across products and easier for consumers to encounter while moving within the same branded environment. These properties position digital food interfaces as an enabling and scaling infrastructure for product-level disclosure and potential cross-product transfer (Olzenak et al., 2020; Pomeranz et al., 2022; Sharib et al., 2024; Volpe et al., 2026; Yang et al., 2025). At the same time, greater disclosure capacity does not ensure that information will be perceived as transparent or decision-relevant. Product information acquires broader meaning only when consumers can locate, interpret, and attribute it to an intentional communication choice by the brand (Cambier & Poncin, 2020; Montecchi et al., 2024; Sansome et al., 2024, 2025). This explains why digital architecture is theoretically consequential without treating digitality itself as an independent psychological mechanism.

Third, the findings refine the theoretical status of voluntary negative-attribute disclosure. Signaling and two-sided communication research suggests that unfavorable information may appear credible when its disclosure is difficult to reconcile with uniformly self-serving persuasion (Connelly et al., 2011; Eisend, 2006; Fennis & Stroebe, 2014; Park & Yi, 2023). Consistent with this reasoning, voluntary negative-attribute disclosure produced more favorable brand trust formation than self-promotion and influenced responses beyond the disclosed product. Comparable downstream outcomes following voluntary negative-attribute disclosure and objective description coexisted with a clearer trust formation pattern for voluntary negative-attribute disclosure. It generated significantly higher cognitive trust than objective description and, relative to self-promotion, increased both cognitive and affective trust. This pattern indicates that voluntarily foregrounding an unfavorable attribute can give a product-level disclosure stronger brand-level meaning by shaping judgments of the brand as a reliable and consumer-oriented information provider. Reduced promotional selectivity offers one plausible explanation for this trust formation advantage and provides a focused direction for future research.

Fourth, the research advances trust transfer theory by distinguishing cognitive trust from affective trust as related but empirically distinguishable brand-level pathways. Prior trust research defines cognitive trust in terms of reliability, integrity, and dependability, whereas affective trust reflects sincerity, benevolence, and relational concern (Johnson & Grayson, 2005; Mayer et al., 1995). The present findings show that a single product-level disclosure can support both forms of brand trust and that both can transmit effects to another product. Study 2 further shows that these pathways differ in persistence when the disclosed drawback violates category expectations. Unexpected information attracts attention and encourages interpretive and attributional processing (Folkes, 1984; Meyers-Levy & Tybout, 1989; Friestad & Wright, 1994). In the present setting, high unexpectedness weakened both trust pathways, but only the affective pathway remained reliably operative. The theoretical contribution lies in the greater persistence of affective trust when forming a stable judgment of brand reliability becomes more difficult.

Finally, the research distinguishes the formation of brand trust from the translation of that trust into specific target-product responses. Brand extension research treats product fit as a basis for judging whether associations formed around one offering are relevant to another (Aaker & Keller, 1990; Bridges et al., 2000; C. Peng et al., 2023; Völckner & Sattler, 2006; Biallowons et al., 2026). The present findings add that the implications of low fit depend partly on the outcome being explained. Target-product trust is a relatively proximal judgment requiring confidence in the reliability of Product B itself. Purchase intention is a more distal and integrative response that can incorporate brand-level trust alongside preferences, value, need, and other choice considerations (Kim et al., 2008; Lim et al., 2006). Accordingly, under low fit, both cognitive and affective brand trust remained relevant to purchase intention while neither reliably established target-product trust. Because the formal indices did not demonstrate significant differences between fit conditions, these findings do not establish product fit as a confirmed selector of competing psychological routes. They instead identify a theoretically informative conditional configuration showing that the same brand-level trust can translate differently depending on what consumers are asked to judge.

### 6.3. Managerial Implications

The findings first suggest that managers should evaluate voluntary negative-attribute disclosure at multiple levels rather than relying on a single purchase-related indicator. Across Studies 1 and 2, the cross-product effect was more consistently expressed in target-product trust than in purchase intention. Managers should therefore monitor cognitive and affective trust in the brand, trust in other products, purchase intention, and actual conversion as distinct outcomes. This distinction is especially important for portfolio-based brands because a disclosure concerning one offering may strengthen confidence in another offering before producing an observable change in purchasing behavior. A suitable evaluation dashboard should consequently track brand trust formation, target-product responses, and behavioral outcomes separately across products and over time.

Second, managers and platform designers should use the capabilities of digital interfaces to make voluntary negative-attribute disclosure visible, interpretable, and useful at the point of choice. Decision-relevant attributes—such as sugar, sodium, caffeine, calories, and other nutrition-related information—can be placed within product pages, customization modules, and portfolio navigation rather than being confined to separate technical documents. The disclosure format should remain consistent across comparable products, use understandable units and labels, and allow consumers to connect the information directly to their ordering decisions (Olzenak et al., 2020; Pomeranz et al., 2022; Sharib et al., 2024). HEYTEA provides a concrete illustration: its electronic ingredient list, launched in 2023, draws on nutritional assessments covering caloric content, caffeine, total sugars, and related components to communicate product information to consumers digitally (SGS, 2024). This type of implementation demonstrates how a brand can integrate decision-relevant product information into the same digital environment in which consumers browse and choose products.

Third, the results help managers distinguish the trust-building value of voluntary negative-attribute disclosure from straightforward factual information provision. Even when its immediate downstream outcomes resemble those observed after an objective description, voluntary negative-attribute disclosure offers a clearer basis for brand trust formation. In Study 1, it generated significantly higher cognitive trust than objective description and, relative to self-promotion, increased both cognitive and affective trust. Managers whose communication objectives include strengthening perceptions of brand reliability, honesty, and consumer orientation may therefore benefit from voluntarily foregrounding an unfavorable but decision-relevant attribute. Such disclosure should form part of a stable communication policy applied consistently across products and occasions so that consumers can recognize it as an enduring feature of how the brand communicates.

Finally, the application of voluntary negative-attribute disclosure requires a disciplined assessment of attribute type and portfolio consequences. The present evidence is most directly relevant to accurate, decision-relevant, relatively minor or moderate, and non-safety-critical attributes such as calorie, sugar, sodium, or caffeine content. Severe defects, contamination risks, major allergen hazards, and other safety-critical information should be managed primarily through safety and compliance procedures. Before adopting voluntary negative-attribute disclosure across a portfolio, managers should assess responses to both the disclosed Product A and other products under the brand, including product trust, choice, purchasing behavior, and profitability. Jointly monitoring focal-product costs and cross-product benefits provides the evidence needed to determine whether a disclosure strategy creates value at the portfolio level.

### 6.4. Limitations and Future Research

First, the present experiments were designed to identify cross-product consequences and therefore measured responses to Product B rather than outcomes for the disclosed Product A. A complete assessment of voluntary negative-attribute disclosure requires both sides of this trade-off. Future research should measure Product A trust, evaluation, choice, and purchase intention together with the corresponding Product B outcomes in the same design while also manipulating the severity and safety relevance of the disclosed attribute. Such designs would reveal when cross-product benefits accompany, offset, or fall short of focal-product costs and would permit a direct estimate of the resulting portfolio-level net effect.

Second, Study 1 indicates that voluntary negative-attribute disclosure has a distinctive capacity to form brand trust. Although its downstream outcomes were statistically comparable with those following objective description, voluntary negative-attribute disclosure generated higher cognitive trust and, relative to self-promotion, increased both cognitive and affective trust. Future research should identify which features of voluntary negative-attribute disclosure produce this stronger trust formation pattern. One approach would be to manipulate information valence and promotional selectivity independently while directly measuring perceived communication diagnosticity, disclosure motive, persuasion intent, and consumer orientation. This design would clarify how consumers convert a product-level disclosure into a brand-level judgment and establish the explanatory roles of these candidate processes (Darke & Ritchie, 2007; Friestad & Wright, 1994).

Third, the experiments captured immediate responses following a single disclosure episode and relied on self-reported target-product trust and purchase intention. Future studies should use delayed- and repeated-exposure designs to determine whether disclosure-based trust accumulates, stabilizes, decays, or reverses over time. They should also incorporate behavioral indicators—such as information search, product comparison, cart addition, actual choice, repeat purchase, and platform log data—to connect trust formation with observable consumer behavior (Alkhunein et al., 2024; Kim et al., 2008; M. Peng et al., 2021).

Fourth, the experiments used a conspicuous, front-facing disclosure module to create a clear manipulation within a controlled digital interface. This format represents one practically relevant implementation, while digital food environments vary in the color, size, placement, and default visibility of product information, as well as in whether information appears directly or through expandable and secondary pages. Future research should systematically manipulate disclosure salience and placement, compare default-expanded with consumer-initiated formats, and test these designs in operational ordering platforms. Such research would establish how interface prominence shapes attention, brand trust formation, and cross-product transfer.

Fifth, the no-prior-Product-A-exposure condition provided a baseline without prior brand interaction, whereas the objective-description condition controlled for Product A exposure without an additional evaluative module. Future research could add a visually matched neutral-module condition to separate the effects of module presence and salience from the effects of disclosure content. A fuller design could also manipulate the distance between Products A and B across several product pairs rather than relying on a limited set of food and lifestyle products. This would separate the contribution of prior brand interaction from the contribution of a particular disclosure and provide a more systematic account of how product relatedness shapes trust translation.

Finally, the studies used Chinese online participants, fictitious brands, and food-related digital interfaces. Future research should examine real brands, diverse consumer populations, and institutional settings in which disclosure expectations and regulations differ. It should also distinguish voluntary foregrounding from mandatory compliance and compare digital interfaces with physical packages, printed menus, in-store displays, and salesperson communication. Extending the framework to markets characterized by difficult-to-verify attributes—such as second-hand vehicles, collectibles, and other credence-oriented products—would help establish where voluntary disclosure is particularly informative and where regulatory disclosure plays the dominant role in consumer inference (Maganja et al., 2023; Pomeranz et al., 2022; Sharib et al., 2024).

## 7. Conclusions

This research examined whether voluntary negative-attribute disclosure in a digital food interface can shape brand-level trust and influence responses to another product under the same brand. Across three online experiments, voluntary negative-attribute disclosure produced a more favorable cross-product pattern than self-promotion and, where included, no prior Product A exposure, with the evidence more consistent for target-product trust than for purchase intention. Cognitive trust and affective trust operated as parallel brand-level pathways through which a product-level disclosure acquired broader meaning for the brand. The findings further showed that unexpectedness weakened both pathways, while the affective trust pathway remained operative under high unexpectedness. Under low product fit, both trust pathways supported purchase intention but did not reliably translate into target-product trust. Together, these findings show that a disclosure concerning one product can shape how consumers evaluate the brand as an information provider and can influence responses beyond the disclosed product. For managers, voluntary negative-attribute disclosure may therefore serve as a trust-building communication approach when the information is accurate, decision-relevant, and presented clearly at the point of choice. More fundamentally, the findings show how voluntarily revealing an unfavorable product attribute can transform a product-level disclosure into brand-level trust that shapes consumers’ responses to other products under the same brand.

## Figures and Tables

**Table 1:** Demographic characteristics of participants in Study 1 (N = 196).

Characteristics	Category	Frequency	Percentage (%)
Gender	Male	85	43.4
	Female	111	56.6
Age	18–25	46	23.5
	26–35	94	48.0
	36–45	29	14.8
	Above 45	27	13.8
Monthly income	Less than 1000	4	2.0
	1000–3000	14	7.1
	3001–5000	27	13.8
	5001–8000	85	43.4
	More than 8000	66	33.7
Education level	Middle school or below	3	1.5
	High school	5	2.6
	Bachelor’s degree	165	84.2
	Master’s degree	18	9.2
	Ph.D. or above	5	2.6
Fast-food frequency (per week)	Less than 1	4	2.0
	2–3	59	30.1
	4–5	70	35.7
	6–7	40	20.4
	More than 7	23	11.7

**Table 2:** Covariate-adjusted planned comparisons in Study 1.

Outcome	Comparison	B	Robust SE	95% CI for B	*p*	g_adj	95% CI for g_adj	Holm *p*
Target-product trust	VND–self-promotion	0.345	0.136	[0.076, 0.613]	0.012	0.519	[0.115, 0.923]	0.037
	VND–objective description	−0.007	0.108	[−0.220, 0.206]	0.951	−0.010	[−0.331, 0.311]	0.951
	VND–no prior Product A exposure	0.278	0.133	[0.015, 0.541]	0.038	0.418	[0.022, 0.814]	0.077
Purchase intention	VND–self-promotion	0.273	0.154	[−0.030, 0.575]	0.078	0.384	[−0.043, 0.810]	0.233
	VND–objective description	−0.016	0.117	[−0.247, 0.215]	0.893	−0.022	[−0.348, 0.303]	0.893
	VND–no prior Product A exposure	0.159	0.139	[−0.115, 0.433]	0.253	0.224	[−0.161, 0.609]	0.506

Note. All models controlled for gender, brand familiarity, and health consciousness; complete coefficients for these covariates are reported in Appendix A (Table A1). VND = voluntary negative-attribute disclosure. g_adj denotes the covariate-adjusted standardized contrast. Holm-adjusted *p*-values constitute sensitivity analyses within each outcome.

**Table 3:** PROCESS Model 4 results for voluntary negative-attribute disclosure versus self-promotion in Study 1.

(**A**). Regression paths, total effects, and direct effects						
**Outcome Equation**	**Effect**	**B**	**SE**	**t**	***p***	**95% CI**
Cognitive trust	VND → cognitive trust	0.656	0.152	4.31	<0.001	[0.354, 0.958]
Affective trust	VND → affective trust	0.468	0.145	3.23	0.002	[0.180, 0.756]
Purchase intention	Cognitive trust → purchase intention	0.364	0.111	3.27	0.002	[0.143, 0.585]
	Affective trust → purchase intention	0.404	0.117	3.46	<0.001	[0.172, 0.636]
	Total effect of VND	0.360	0.159	2.26	0.026	[0.043, 0.676]
	Direct effect of VND	−0.068	0.134	−0.51	0.613	[−0.333, 0.198]
Target-product trust	Cognitive trust → target-product trust	0.296	0.100	2.97	0.004	[0.098, 0.494]
	Affective trust → target-product trust	0.385	0.105	3.68	<0.001	[0.177, 0.592]
	Total effect of VND	0.407	0.142	2.86	0.005	[0.125, 0.689]
	Direct effect of VND	0.033	0.120	0.27	0.786	[−0.205, 0.270]
(**B**). Bootstrap indirect effects						
**Outcome**	**Indirect Effect**	**B**	**BootSE**	**95% Bootstrap CI**		
Purchase intention	Total indirect effect	0.428	0.145	[0.152, 0.728]		
	Via cognitive trust	0.239	0.111	[0.039, 0.477]		
	Via affective trust	0.189	0.088	[0.047, 0.386]		
Target-product trust	Total indirect effect	0.374	0.120	[0.154, 0.616]		
	Via cognitive trust	0.194	0.079	[0.044, 0.357]		
	Via affective trust	0.180	0.087	[0.045, 0.376]		

Note. N = 99. Voluntary negative-attribute disclosure was coded 1, and self-promotion was coded 0. All models controlled for gender, brand familiarity, and health consciousness; complete coefficients for these covariates are reported in Appendix A (Table A1). Indirect effects were estimated using 5000 percentile bootstrap samples.

**Table 4:** Demographic characteristics of participants in Study 2 (N = 434).

Characteristics	Category	Frequency	Percentage (%)
Gender	Male	193	44.5
	Female	241	55.5
Age	18–25	113	26.0
	26–35	202	46.5
	36–45	63	14.5
	Above 45	56	12.9
Monthly income	Less than 1000	10	2.3
	1000–3000	36	8.3
	3001–5000	55	12.7
	5001–8000	183	42.2
	More than 8000	150	34.6
Education level	Middle school or below	4	0.9
	High school	12	2.8
	Bachelor’s degree	359	82.7
	Master’s degree	48	11.1
	Ph.D. or above	11	2.5
Fast-food frequency (per week)	Less than 1	7	1.6
	2–3	102	23.5
	4–5	146	33.6
	6–7	108	24.9
	More than 7	71	16.4

**Table 5:** Covariate-adjusted planned comparisons in Study 2.

Outcome	Comparison	B	Robust SE	95% CI for B	*p*	g_adj	95% CI for g_adj	Holm *p*
Target-product trust	VND–self-promotion	0.227	0.089	[0.053, 0.401]	0.011	0.344	[0.080, 0.607]	0.032
	VND–objective description	0.049	0.082	[−0.112, 0.209]	0.552	0.074	[−0.169, 0.317]	0.552
	VND–no prior Product A exposure	0.225	0.090	[0.049, 0.401]	0.012	0.341	[0.074, 0.607]	0.032
Purchase intention	VND–self-promotion	0.213	0.098	[0.021, 0.406]	0.030	0.299	[0.029, 0.568]	0.090
	VND–objective description	−0.048	0.083	[−0.211, 0.116]	0.568	−0.066	[−0.295, 0.162]	0.841
	VND–no prior Product A exposure	0.076	0.094	[−0.109, 0.262]	0.420	0.106	[−0.153, 0.366]	0.841

Note. All models controlled for gender, brand familiarity, and health consciousness; complete coefficients for these covariates are reported in Appendix A (Table A2). VND = voluntary negative-attribute disclosure. g_adj denotes the covariate-adjusted standardized contrast. Holm-adjusted *p*-values constitute sensitivity analyses within each outcome.

**Table 6:** PROCESS Model 7 results for Study 2.

(**A**). First-stage moderation						
**Outcome Equation**	**Effect**	**B**	**SE**	**t**	***p***	**95% CI**
Cognitive trust	Disclosure strategy × unexpectedness	−0.541	0.225	−2.41	0.017	[−0.984, −0.098]
	Effect of VND under low unexpectedness	0.636	0.165	3.84	<0.001	[0.310, 0.962]
	Effect of VND under high unexpectedness	0.095	0.151	0.63	0.529	[−0.202, 0.392]
Affective trust	Disclosure strategy × unexpectedness	−0.386	0.179	−2.16	0.032	[−0.738, −0.034]
	Effect of VND under low unexpectedness	0.620	0.132	4.72	<0.001	[0.361, 0.880]
	Effect of VND under high unexpectedness	0.234	0.120	1.96	0.052	[−0.002, 0.471]
(**B**). Conditional indirect effects						
**Outcome**	**Mediator**	**Unexpectedness**	**Indirect Effect**	**BootSE**	**95% Bootstrap CI**	
Purchase intention	Cognitive trust	Low	0.188	0.081	[0.059, 0.375]	
	Cognitive trust	High	0.028	0.049	[−0.058, 0.141]	
	Affective trust	Low	0.292	0.094	[0.128, 0.494]	
	Affective trust	High	0.110	0.058	[0.007, 0.235]	
Target-product trust	Cognitive trust	Low	0.205	0.069	[0.091, 0.356]	
	Cognitive trust	High	0.031	0.051	[−0.059, 0.143]	
	Affective trust	Low	0.220	0.076	[0.089, 0.381]	
	Affective trust	High	0.083	0.043	[0.006, 0.177]	
(**C**). Indices of moderated mediation						
**Outcome**	**Mediator**	**Index**	**BootSE**	**95% Bootstrap CI**		
Purchase intention	Cognitive trust	−0.160	0.084	[−0.356, −0.026]		
	Affective trust	−0.182	0.095	[−0.385, −0.018]		
Target-product trust	Cognitive trust	−0.174	0.076	[−0.342, −0.043]		
	Affective trust	−0.137	0.074	[−0.297, −0.013]		

Note. N = 218. Voluntary negative-attribute disclosure was coded 1 and self-promotion was coded 0. Low unexpectedness was coded 0 and high unexpectedness was coded 1. All models controlled for gender, brand familiarity, and health consciousness; complete coefficients for these covariates are reported in Appendix A (Table A2). Conditional indirect effects and indices of moderated mediation were estimated using 5000 percentile bootstrap samples. VND = voluntary negative-attribute disclosure.

**Table 7:** Demographic characteristics of respondents in Study 3 (N = 188).

Characteristics	Category	Frequency	Percentage (%)
Gender	Male	91	48.4
	Female	97	51.6
Age	18–25	43	22.9
	26–35	102	54.3
	36–45	24	12.8
	Above 45	19	10.1
Monthly income	Less than 1000	0	0.0
	1000–3000	15	8.0
	3001–5000	23	12.2
	5001–8000	72	38.3
	More than 8000	78	41.5
Education level	Middle school or below	0	0.0
	High school	8	4.3
	Bachelor’s degree	142	75.5
	Master’s degree	32	17.0
	Ph.D. or above	6	3.2
Fast-food frequency (per week)	Less than 1	8	4.3
	2–3	56	29.8
	4–5	79	42.0
	6–7	22	11.7
	More than 7	23	12.2

**Table 8:** PROCESS Model 14 results for Study 3.

(**A**). Second-stage moderation						
**Outcome**	**Interaction**	**B**	**SE**	**t**	***p***	**95% CI**
Purchase intention	Cognitive trust × product fit	−0.123	0.234	−0.53	0.600	[−0.586, 0.340]
	Affective trust × product fit	−0.255	0.246	−1.04	0.302	[−0.741, 0.232]
Target-product trust	Cognitive trust × product fit	0.111	0.264	0.42	0.673	[−0.411, 0.633]
	Affective trust × product fit	0.541	0.277	1.95	0.053	[−0.008, 1.090]
(**B**). Conditional indirect effects						
**Outcome**	**Mediator**	**Product Fit**	**Indirect Effect**	**BootSE**	**95% Bootstrap CI**	
Purchase intention	Cognitive trust	Low	0.337	0.172	[0.056, 0.721]	
	Cognitive trust	High	0.260	0.107	[0.072, 0.490]	
	Affective trust	Low	0.283	0.133	[0.051, 0.565]	
	Affective trust	High	0.153	0.106	[0.001, 0.415]	
Target-product trust	Cognitive trust	Low	0.200	0.182	[−0.124, 0.603]	
	Cognitive trust	High	0.270	0.110	[0.082, 0.508]	
	Affective trust	Low	−0.101	0.164	[−0.434, 0.223]	
	Affective trust	High	0.176	0.116	[0.013, 0.468]	
(**C**). Indices of moderated mediation						
**Outcome**	**Mediator**	**Index**	**BootSE**	**95% Bootstrap CI**		
Purchase intention	Cognitive trust	−0.077	0.167	[−0.431, 0.228]		
	Affective trust	−0.130	0.134	[−0.386, 0.151]		
Target-product trust	Cognitive trust	0.070	0.203	[−0.343, 0.477]		
	Affective trust	0.277	0.212	[−0.082, 0.751]		

Note. N = 127. Voluntary negative-attribute disclosure was coded 1, and self-promotion was coded 0. Low product fit was coded 0, and high product fit was coded 1. All models controlled for gender, brand familiarity, and health consciousness; complete coefficients for these covariates are reported in Appendix A (Table A3). Conditional indirect effects and indices of moderated mediation were estimated using 5000 percentile bootstrap samples.

## Data Availability

The raw data supporting the conclusions of this article will be made available by the authors on request.

## Appendix A

Table A1, Table A2 and Table A3 report the coefficients for gender, brand familiarity, and health consciousness from the main effect and PROCESS models summarized in the main text. Focal experimental effects, conditional indirect effects, moderated mediation indices, and Holm-adjusted sensitivity results are reported in the corresponding main-text tables and are not repeated here.

**Table A1:** Complete covariate coefficients for Study 1 models.

Analysis	Equation	Gender	Brand Familiarity	Health Consciousness
H1 main effect model	Purchase intention	B = 0.097 (0.102), t = 0.95, *p* = 0.341	B = 0.117 (0.027), t = 4.30, *p* < 0.001	B = −0.079 (0.039), t = −1.99, *p* = 0.048
H1 main effect model	Target-product trust	B = 0.048 (0.098), t = 0.49, *p* = 0.624	B = 0.081 (0.027), t = 2.96, *p* = 0.003	B = −0.053 (0.035), t = −1.49, *p* = 0.138
VND vs. self-promotion	Cognitive trust	B = 0.130 (0.158), t = 0.82, *p* = 0.413	B = −0.207 (0.078), t = −2.67, *p* = 0.009	B = −0.011 (0.067), t = −0.17, *p* = 0.869
VND vs. self-promotion	Affective trust	B = 0.116 (0.150), t = 0.77, *p* = 0.442	B = −0.223 (0.074), t = −3.02, *p* = 0.003	B = −0.004 (0.064), t = −0.06, *p* = 0.956
VND vs. self-promotion	Purchase intention	B = 0.099 (0.127), t = 0.78, *p* = 0.438	B = 0.089 (0.065), t = 1.36, *p* = 0.176	B = −0.092 (0.054), t = −1.71, *p* = 0.090
VND vs. self-promotion	Purchase intention (total effect model)	B = 0.193 (0.165), t = 1.17, *p* = 0.246	B = −0.076 (0.081), t = −0.94, *p* = 0.350	B = −0.097 (0.070), t = −1.39, *p* = 0.168
VND vs. self-promotion	Target-product trust	B = 0.002 (0.114), t = 0.02, *p* = 0.985	B = 0.118 (0.059), t = 2.01, *p* = 0.047	B = −0.079 (0.048), t = −1.65, *p* = 0.103
VND vs. self-promotion	Target-product trust (total effect model)	B = 0.085 (0.147), t = 0.58, *p* = 0.565	B = −0.029 (0.072), t = −0.40, *p* = 0.688	B = −0.084 (0.062), t = −1.34, *p* = 0.184
VND vs. objective description	Cognitive trust	B = 0.121 (0.146), t = 0.83, *p* = 0.407	B = 0.093 (0.075), t = 1.24, *p* = 0.218	B = 0.274 (0.070), t = 3.90, *p* < 0.001
VND vs. objective description	Affective trust	B = 0.381 (0.133), t = 2.87, *p* = 0.005	B = 0.052 (0.068), t = 0.77, *p* = 0.445	B = 0.252 (0.064), t = 3.93, *p* < 0.001
VND vs. objective description	Purchase intention	B = 0.205 (0.117), t = 1.75, *p* = 0.084	B = 0.086 (0.057), t = 1.50, *p* = 0.138	B = 0.062 (0.058), t = 1.07, *p* = 0.287
VND vs. objective description	Purchase intention (total effect model)	B = 0.334 (0.118), t = 2.82, *p* = 0.006	B = 0.104 (0.061), t = 1.71, *p* = 0.090	B = 0.149 (0.057), t = 2.61, *p* = 0.011
VND vs. objective description	Target-product trust	B = −0.015 (0.108), t = −0.14, *p* = 0.889	B = 0.015 (0.053), t = 0.29, *p* = 0.774	B = −0.022 (0.054), t = −0.42, *p* = 0.675
VND vs. objective description	Target-product trust (total effect model)	B = 0.077 (0.110), t = 0.70, *p* = 0.487	B = 0.040 (0.057), t = 0.70, *p* = 0.486	B = 0.068 (0.053), t = 1.28, *p* = 0.203
Objective description vs. self-promotion	Cognitive trust	B = 0.237 (0.190), t = 1.25, *p* = 0.216	B = 0.267 (0.111), t = 2.40, *p* = 0.018	B = 0.313 (0.099), t = 3.18, *p* = 0.002
Objective description vs. self-promotion	Affective trust	B = 0.489 (0.178), t = 2.75, *p* = 0.007	B = 0.086 (0.104), t = 0.82, *p* = 0.411	B = 0.320 (0.092), t = 3.48, *p* < 0.001
Objective description vs. self-promotion	Purchase intention	B = −0.100 (0.131), t = −0.76, *p* = 0.447	B = 0.096 (0.076), t = 1.26, *p* = 0.212	B = 0.056 (0.069), t = 0.81, *p* = 0.421
Objective description vs. self-promotion	Purchase intention (total effect model)	B = 0.151 (0.159), t = 0.95, *p* = 0.345	B = 0.167 (0.092), t = 1.80, *p* = 0.075	B = 0.239 (0.082), t = 2.91, *p* = 0.004
Objective description vs. self-promotion	Target-product trust	B = −0.112 (0.128), t = −0.87, *p* = 0.386	B = −0.003 (0.075), t = −0.05, *p* = 0.964	B = 0.018 (0.068), t = 0.26, *p* = 0.795
Objective description vs. self-promotion	Target-product trust (total effect model)	B = 0.030 (0.146), t = 0.20, *p* = 0.840	B = 0.097 (0.085), t = 1.13, *p* = 0.261	B = 0.163 (0.076), t = 2.15, *p* = 0.034

Note. Entries report unstandardized coefficients. Parentheses contain standard errors. All PROCESS analyses used 5000 percentile bootstrap samples. The exploratory contrasts were conducted after the comparable downstream outcomes for VND and objective description were observed. VND = voluntary negative-attribute disclosure.

**Table A2:** Complete covariate coefficients for Study 2 models.

Analysis	Equation	Gender	Brand Familiarity	Health Consciousness
H1 main effect model	Purchase intention	B = 0.062 (0.070), t = 0.88, *p* = 0.381	B = 0.148 (0.020), t = 7.36, *p* < 0.001	B = −0.014 (0.027), t = −0.50, *p* = 0.617
H1 main effect model	Target-product trust	B = 0.085 (0.066), t = 1.30, *p* = 0.195	B = 0.077 (0.019), t = 4.13, *p* < 0.001	B = −0.034 (0.023), t = −1.50, *p* = 0.134
VND vs. self-promotion (PROCESS Model 7)	Cognitive trust	B = 0.212 (0.113), t = 1.87, *p* = 0.062	B = 0.103 (0.060), t = 1.71, *p* = 0.089	B = 0.181 (0.057), t = 3.18, *p* = 0.002
VND vs. self-promotion (PROCESS Model 7)	Affective trust	B = 0.179 (0.090), t = 1.99, *p* = 0.047	B = 0.000 (0.048), t = 0.01, *p* = 0.994	B = 0.059 (0.045), t = 1.31, *p* = 0.192
VND vs. self-promotion (PROCESS Model 7)	Purchase intention	B = −0.085 (0.085), t = −0.99, *p* = 0.322	B = 0.015 (0.045), t = 0.34, *p* = 0.736	B = −0.002 (0.043), t = −0.04, *p* = 0.967
VND vs. self-promotion (PROCESS Model 7)	Target-product trust	B = −0.003 (0.074), t = −0.03, *p* = 0.973	B = −0.028 (0.039), t = −0.72, *p* = 0.475	B = −0.026 (0.037), t = −0.71, *p* = 0.476

Note. Entries report unstandardized coefficients. Parentheses contain standard errors. Low unexpectedness was coded 0 and high unexpectedness was coded 1 in PROCESS Model 7. VND = voluntary negative-attribute disclosure.

**Table A3:** Complete covariate coefficients for Study 3 models.

Analysis	Equation	Gender	Brand Familiarity	Health Consciousness
VND vs. self-promotion (PROCESS Model 14)	Cognitive trust	B = −0.140 (0.158), t = −0.89, *p* = 0.377	B = 0.163 (0.091), t = 1.78, *p* = 0.077	B = 0.045 (0.075), t = 0.60, *p* = 0.551
VND vs. self-promotion (PROCESS Model 14)	Affective trust	B = −0.096 (0.148), t = −0.65, *p* = 0.516	B = 0.279 (0.085), t = 3.28, *p* = 0.001	B = 0.158 (0.070), t = 2.24, *p* = 0.027
VND vs. self-promotion (PROCESS Model 14)	Purchase intention	B = 0.021 (0.124), t = 0.17, *p* = 0.868	B = 0.042 (0.074), t = 0.57, *p* = 0.569	B = 0.065 (0.061), t = 1.06, *p* = 0.290
VND vs. self-promotion (PROCESS Model 14)	Target-product trust	B = −0.139 (0.140), t = −0.99, *p* = 0.322	B = −0.060 (0.083), t = −0.72, *p* = 0.473	B = 0.044 (0.069), t = 0.64, *p* = 0.526

Note. Entries report unstandardized coefficients. Parentheses contain standard errors. Low product fit was coded 0 and high product fit was coded 1 in PROCESS Model 14. VND = voluntary negative-attribute disclosure.
